# Pistil Transcriptome Analysis to Disclose Genes and Gene Products Related to Aposporous Apomixis in *Hypericum perforatum* L.

**DOI:** 10.3389/fpls.2017.00079

**Published:** 2017-02-01

**Authors:** Giulio Galla, Sara Zenoni, Linda Avesani, Lothar Altschmied, Paride Rizzo, Timothy F. Sharbel, Gianni Barcaccia

**Affiliations:** ^1^Laboratory of Genomics, Department of Agronomy, Food, Natural Resources, Animals and Environment, University of PadovaPadova, Italy; ^2^Department of Biotechnology, University of VeronaVerona, Italy; ^3^Department of Molecular Genetics, Leibniz Institute of Plant Genetics and Crop Plant ResearchGatersleben, Germany; ^4^Department of Breeding Research, Leibniz Institute of Plant Genetics and Crop Plant ResearchGatersleben, Germany

**Keywords:** *Hypericum perforatum*, sexual reproduction, aposporous apomixis, microarray

## Abstract

Unlike sexual reproduction, apomixis encompasses a number of reproductive strategies, which permit maternal genome inheritance without genetic recombination and syngamy. The key biological features of apomixis are the circumvention of meiosis (i.e., apomeiosis), the differentiation of unreduced embryo sacs and egg cells, and their autonomous development in functional embryos through parthenogenesis, and the formation of viable endosperm either via fertilization-independent means or following fertilization with a sperm cell. Despite the importance of apomixis for breeding of crop plants and although much research has been conducted to study this process, the genetic control of apomixis is still not well understood. *Hypericum perforatum* is becoming an attractive model system for the study of aposporous apomixis. Here we report results from a global gene expression analysis of *H. perforatum* pistils collected from sexual and aposporous plant accessions for the purpose of identifying genes, biological processes and molecular functions associated with the aposporous apomixis pathway. Across two developmental stages corresponding to the expression of aposporous apomeiosis and parthenogenesis in ovules, a total of 224 and 973 unigenes were found to be significantly up- and down-regulated with a fold change ≥ 2 in at least one comparison, respectively. Differentially expressed genes were enriched for multiple gene ontology (GO) terms, including cell cycle, DNA metabolic process, and single-organism cellular process. For molecular functions, the highest scores were recorded for GO terms associated with DNA binding, DNA (cytosine-5-)-methyltransferase activity and heterocyclic compound binding. As deregulation of single components of the sexual developmental pathway is believed to be a trigger of the apomictic reproductive program, all genes involved in sporogenesis, gametogenesis and response to hormonal stimuli were analyzed in great detail. Overall, our data suggest that phenotypic expression of apospory is concomitant with the modulation of key genes involved in the sexual reproductive pathway. Furthermore, based on gene annotation and co-expression, we underline a putative role of hormones and key actors playing in the RNA-directed DNA methylation pathway in regulating the developmental changes occurring during aposporous apomixis in *H. perforatum*.

## Introduction

Apomixis defines a number of reproductive strategies, which, unlike sexual reproduction, permit the inheritance of the maternal genome without genetic recombination and syngamy. This strategy of asexual reproduction is documented in more than 120 angiosperm genera (Carman, 1997); however, no major seed crop species are apomictic, and attempts to introduce the apomixis trait into crop plants from apomictic relatives via conventional breeding schemes have been largely unsuccessful (Barcaccia and Albertini, 2013). This asexual mode of seed formation is a trait with enormous economic and social potential in agriculture, as genetically fixing highly complex genotypes, including hybrid cultivars, through apomixis would have tremendous advantages in plant breeding, biomass and seed production (Calzada et al., 1996).

The key biological features of apomixis are the circumvention of meiosis (i.e., apomeiosis), the differentiation of unreduced embryo sacs and egg cells, and their autonomous development in functional embryos without fertilization (i.e., parthenogenesis), and the formation of viable endosperm either via fertilization-independent means or following fertilization with a sperm cell (Koltunow and Grossniklaus, 2003). In particular, gametophytic apomixis occurs with the parthenogenic development of unreduced egg cells from apomeiotic embryo sacs that can originate from a nucellar somatic cell (i.e., apospory) or a megaspore mother cell with no, or modified, meiosis (i.e., diplospory). These features deviate from sexuality as the capability to develop an embryo sac is strictly restricted to the reduced functional megaspore deriving from meiosis, and failure of the meiotic programme naturally results in the abortion of the ovule. While polyploidy does not appear to be absolutely required for the expression of apomixis, plant species reproducing through gametophytic apomixis are very often polyploids and/or hybrids in nature (Bicknell and Koltunow, 2004). Hence, it has been suggested that apomixis might be a consequence of epigenomic shock resulting from interspecific hybridization and polyploidization, which induce transcriptome changes and the deregulation of reproductive development (Garcia-Aguilar et al., 2010). Several lines of evidence suggest that such dramatic shifts in the reproductive process could rely on spatial and/or temporal changes in the expression of sexual pathway-related genes (Grimanelli et al., 2003; Sharbel et al., 2010).

Plants reproducing via aposporous apomixis (Nogler, 1984) possess the ability to differentiate viable unreduced embryo sacs from somatic cells of the ovule (i.e., aposporous initial cells). In principle, these cells are somatic cells belonging to the nucellus, which change their fate by being able to mitotically divide and develop functional embryo sacs by mimicking sexual gametogenesis development (Barcaccia et al., 2007; Galla et al., 2011). Whether the competence to develop functional embryo sacs is retained by all nucellar cells or is restricted to a few nucellar cells physically connected to the meiocytes or the meiospores in the ovule is not yet fully understood.

In recent years, expression studies and the characterization of mutants that are defective in their ability to set seeds has led to the identification of many genes whose expression patterns are necessary for proper ovule development and correct progression of sporogenesis and gametogenesis in model plant species (Christensen et al., 1997; Drews et al., 1998; Drews and Koltunow, 2011). The ability to undergo meiosis and gametogenesis is apparently restricted to particular cell types and is determined by the expression and interaction of mRNAs and small RNAs which are necessary for the proper specification of cell identity within the ovule as well, as for the onset and progression of sporogenesis and gametogenesis (Olmedo-Monfil et al., 2010; Armenta-Medina et al., 2011; Singh et al., 2011; Tucker et al., 2012). Interestingly, Garcia-Aguilar et al. (2010) reported that the DNA methylation pathway active during reproduction is essential for gametophyte development, and observed that loss-of-function mutants for two genes involved in the maintenance of DNA-methylation status produced phenotypes that are strikingly reminiscent of apomictic development, suggesting that DNA methylation in the maize ovule might regulate the transcriptional expression of genes involved in the differentiation between apomixis and sexual reproduction.

Cellular levels of hormones including cytokinins, auxins and polyamines, among others, are also very important for fine-tuning ovule and embryo sac development (Bencivenga et al., 2012; Ceccato et al., 2013; Schmidt et al., 2014). Thus, while cytokinin negatively regulates cell proliferation in the sporophytic tissues surrounding the developing embryo sac (Cheng et al., 2013), auxin biosynthetic and influx genes are necessary for correct growth of the female gametophyte and proper specification of its cell types (Panoli et al., 2015).

*Hypericum perforatum* is an invasive perennial herb that is widely distributed in a variety of habitats and is regarded as a serious weed in many countries (Robson, 2002; Nürk et al., 2013). In recent years, *H. perforatum* has been studied to identify potential genes involved in the biosynthesis of active metabolites (He et al., 2012; Hofrichter et al., 2013). *H. perforatum* reproduces via aposporous apomixis (Noack, 1939; Davis, 1967; Mártonfi et al., 1996; Matzk et al., 2001; Barcaccia et al., 2007; Galla et al., 2011, 2015). As with other asexual plant complexes, apomixis and hybridization are closely linked in *H. perforatum* (Koch et al., 2013), and interestingly, the dosage of genetic factors has been proposed to influence the penetrance of apomixis, as tetraploid and hexaploid genotypes tend to be more apomictic and sexual, respectively, regardless of geographic origin (Molins et al., 2014) Recently, next generation sequencing (NGS) technologies have been used to sequence the flower transcriptomes of obligate sexual and unrelated apomictic *H. perforatum* genotypes (Galla et al., 2015). Here we expand on this work by performing a gene expression analysis of *H. perforatum* pistils collected from sexual and aposporous genotypes for the purpose of identifying genes and gene products, along with biological processes and biosynthetic pathways/networks potentially associated with aposporous apomixis.

## Materials and methods

### Plant materials

*Hypericum perforatum* L. plants from 2 naturally occurring tetraploids (2*n* = 4*x* = 32) and 3 induced tetraploids (2*n* = 4*x* = 32) were used for the microarray approaches (Table 1). For the production of induced tetraploids, plants of the diploid sexual line R1 (reselected from the tetraploid apomictic cultivar “Topaz”) were converted to auto-polyploids by colchicine application, as reported by Schallau et al. (2010). Briefly, seeds were imbibed in water for 24 h, placed on filter paper soaked with an aqueous solution of 0.2% colchicine for 24 h and then planted in soil for germination. The C0 plants that survived this treatment were self-pollinated, and their progenies (C1) were screened for tetraploid plants. Sexual tetraploid C1 and C2 plants were used for further crosses with tetraploid apomictic pollinators and were screened for their reproductive phenotype (Schallau et al., 2010; Molins et al., 2014). The reproductive mode of all *H. perforatum* accessions was estimated by flow cytometric screening of 48 single seeds as described by Matzk et al. (2001).

**Table 1:** **Origins of ***H. perforatum*** samples. For each plant accession, the description, the genealogy, the origin, the ploidy and the degree of apomixis is indicated**.

**Sample ID**	**Accession**	**Description**	**Genealogy**	**Origin**	**Ploidy**	**Apospory**	**Reproduction**
Sex1A-2A	141EU^◦^	Experimental population	4(F12 × No)1a/39	IPK-Gatersleben (D)	*2n* = 4x	<4%	sexual
Sex1B-2B	80EU^◦^	Experimental population	4(F12 × No)1a	IPK-Gatersleben (D)	*2n* = 4x	<4%	sexual
Sex1C-2C	770EU^◦^	Experimental population	4(F12 × No)1a/10	IPK-Gatersleben (D)	*2n* = 4x	<4%	sexual
Apo1A-2A	55EU^◦^	Experimental population	4(F12 × No)1a/8	IPK-Gatersleben (D)	*2n* = 4x	>96%	obligate apomictic
Apo1B-2B	1886US^◦^	Wild population	n.a.	Iron Mountain (USA)	*2n* = 4x	>96%	obligate apomictic
Apo1C^+^	222EU^◦^	Experimental population	4(R1C2xSi)1c/2	IPK-Gatersleben (D)	*2n* = 4x	>96%	obligate apomictic
Apo2C^++^	3348EU^◦^	Wild population	n.a.	Hamburg (D)	*2n* = 4x	>96%	obligate apomictic
Sex	770EU.1^*^	Experimental population	4(F12 × No)1a/10/1	IPK-Gatersleben (D)	*2n* = 4x	<4%	sexual
Sex	770EU.13^*^	Experimental population	4(F12 × No)1a/10/13	IPK-Gatersleben (D)	*2n* = 4x	<4%	sexual
Sex	770EU.18^*^	Experimental population	4(F12 × No)1a/10/18	IPK-Gatersleben (D)	*2n* = 4x	<4%	sexual
Sex	770EU.20^*^	Experimental population	4(F12 × No)1a/10/20	IPK-Gatersleben (D)	*2n* = 4x	<4%	sexual
Sex	770EU.35^*^	Experimental population	4(F12 × No)1a/10/35	IPK-Gatersleben (D)	*2n* = 4x	<4%	sexual
Apo	770EU.39^*^	Experimental population	4(F12 × No)1a/10/39	IPK-Gatersleben (D)	*2n* = 4x	>96%	obligate apomictic
Apo	55.7^*^	Experimental population	4(F12 × No)1a/8/7	IPK-Gatersleben (D)	*2n* = 4x	>96%	obligate apomictic
Apo	HP13.2013^*^	Wild population	n.a.	BL (I)	*2n* = 4x	>96%	obligate apomictic
Apo	Hp1.10.2013^*^	Wild population	n.a.	BL (I)	*2n* = 4x	>96%	obligate apomictic
Apo	Hp1.2013.4^*^	Wild population	n.a.	BL (I)	*2n* = 4x	>96%	obligate apomictic

Apospory expressed as a percentage was determined by flow cytometric screening of 48 single seeds. For details on the origin and composition of experimental populations, please refer to Schallau et al. (2010). Accessions marked with

◦ were used for the microarray expression analysis, while those marked with

* *were used for Real-Time qPCR assays*.

+ *The apomictic accessions 222EU was selected for the flower developmental stage 11*.

++ *The apomictic accessions 3348EU was selected for the flower developmental stage 14*.

Pistils were collected from three apomictic and three sexual plant accessions. In both cases, the developmental stages considered for sampling were bud lengths of approximately 4.0 mm, corresponding to *Arabidopsis* flower stage 11, and bud lengths of approximately 10.0 mm, corresponding to *Arabidopsis* flower developmental stage 14 (Galla et al., 2011).

### Array design, cDNA synthesis and hybridization

Sequences used for the design of 4x180 arrays (Agilent) were previously deposited at DDBJ/EMBL/GenBank under the accession GBXG00000000. The 60,594 tentative consensus sequences were used to design 60-mer oligonucleotide probes for a microarray analysis. Sequences corresponding to transcripts <100 nt in size were removed to reduce the set of target transcripts to 55,682 sequences. The custom microarray with the design ID 065680 was created using eArray, a free Agilent web-based application that enables the creation of custom microarray designs and oligo libraries (https://earray.chem.agilent.com/earray/). For probe design, the Base Composition Methodology option was employed, and no linker sequence was used. Two different probes were designed for each sequence. A total of 111,200 probes were obtained from eArray and were used for creation of the 4x180K array format. Slides with printed arrays were ordered directly from Agilent Technologies (Santa Clara, CA, USA).

### RNA extraction and labelling

Total RNA was extracted from collected pistils using the Spectrum™ Plant Total RNA Kit (Sigma-Aldrich) following the protocol provided by the manufacturer. The eventual contamination of genomic DNA was avoided by using optional DNase I (Sigma-Aldrich) treatment. The abundance and pureness of RNAs were assessed using a NanoDrop 2000c UV-Vis spectrophotometer (Thermo Scientific, Pittsburgh, PA). RNA integrity was evaluated with an Agilent 2100 Bioanalyser with RNA Nanochips (Agilent Technologies, Santa Clara, USA). Only samples with RIN values higher than 7.5 were used in the following procedures. The cRNAs were synthesized from 200 ng of total RNA and labeled with cyanine 3 (Cy3)-CTP fluorescent dye according to the manufacturer's instructions (Agilent Technologies). Aliquots of Cy3-labeled cRNAs (1.65 μg) of each sample were used for subsequent hybridization in a *H. perforatum* custom oligo microarray with the design number 065680 (Agilent Technologies) according to the manufacturer's manual. After hybridization for 17 h at 65°C, slides were washed and scanned with an Agilent Microarray Scanner (G2565CA, Agilent Technologies).

### Statistical analysis

Scanned images were transformed into quantified figures using the Agilent Features extraction software (v11.5), and expression data were normalized based on the 75th percentile. Principal component analyses were carried out using T-MeV v4.9.0 software (http://mev.tm4.org) and by using Manhattan distances. Statistical analyses (*t*-test, unpaired; *p* < 0.01) were carried out using T-MeV v4.9.0 software (http://mev.tm4.org) to define significantly modulated genes between groups of samples. Differentially Expressed Genes (DEGs) were clustered according to their expression patterns in eight groups as reported in Table 2. For each gene and comparison, the fold change (FC) was calculated as the ratio between the average of the normalized expression data measured in three replicates for test and reference samples. For each gene and comparison, the average of the normalized expression data measured for the sexual samples or the average of the normalized expression data measured for the samples corresponding to the earlier developmental stage (floral stage 11) was set as reference (Table 2).

**Table 2:** **Differentially expressed genes**.

**Comparison**	**Biological process**	**DEGs**	**Cluster**	**No**
Sex11^r^ vs. Sex14^t^	sporogenesis and gametogenesis	86	C1 (up)	33
			C2 (down)	53
Apo11^r^ vs. Apo14^t^	sporogenesis, apomeiosis, cell fate changes leading to aposporous initial differentiation and gametogenesis	319	C3 (up)	137
			C4 (down)	182
Sex11^r^ vs. Apo11^t^	sporogenesis, apomeiosis and cell fate changes leading to aposporous initial differentiation	760	C5 (up)	36
			C6 (down)	724
Sex14^r^ vs. Apo14^t^	gametogenesis and pollen perception	32	C7 (up)	18
			C8 (down)	14

*For each comparison the table reports on the main biological process occurring within ovules and the number of identified differentially expressed unigenes (DEGs). Three biological replicates were adopted for both test (t) and reference (r) samples. For each comparison the number of up- and down- regulated unigenes is also indicated*.

### Bioinformatics–array annotation

Annotation of sequences used for the design of 4x180 arrays (Agilent) is described in Galla et al. (2015). Briefly, to annotate all assembled unigenes, a BLASTX-based approach was used to compare the *Hypericum* sequences to the nr database downloaded from the NCBI (http://www.ncbi.nlm.nih.gov/). The following approach was adopted to improve our efficacy in annotating 18,386 unigenes that did not reach significance for the BLAST results in the nr database. Unigenes were aligned to the *Hypericum* genome draft HEID 914233 v1 (Altschmied, personal communication) using BLAT (match ≥ 50 base pairs, fident ≥ 0.97). Additionally, high-quality genomic top hits were selected by preventing the existence of long non-aligned segments on the same side for each unigene and genomic contig. Briefly, a genomic top hits was retained only when the alignment fulfilled the two following criteria (hereafter described for the 5′end of the alignment): (i) 5′ unaligned segment of the genomic contig equal or larger than the 5′ unaligned segment of the unigene only when the 5′ unaligned segment of the unigene was equal or smaller than 10 bp (allowing for an eventually untrimmed adapter sequence at the 5′ end of the unigene) (ii) 3′ unaligned segment of the genomic contig equal or larger than the 3′ unaligned segment of the unigene was allowed only when 3′ unaligned segment of the unigene was equal or smaller than 10 bp (allowing for an eventually untrimmed adapter sequence at the 3′ end of the unigene) or in cases where the unaligned segment of the genomic contig at the 3′ side was smaller than 3 bp. The same criteria were adopted for the 3′ ends of each alignment.

Next, high quality top hits were identified, and genomic sequences upstream and/or downstream of the aligned segments of the genomic top hits with min. 100 and max. 1001 bases of genomic sequence were extracted. All extracted sequences were then used to query the *Arabidopsis thaliana* TAIR10 proteome with BLASTX (*E*-value 1e-10; -seg no; -culling_limit 1). Top hits for genomic sequences flanking the aligned unigenes were then selected for the following annotations.

The GI identifiers of the best BLASTX hits, with *E*-values ≤ 1 E-09 and similarities ≥ 70%, were mapped to the UniProtKB protein database (http://www.uniprot.org/) to extract Gene Ontology (GO, http://www.geneontology.org/) terms for further functional annotations. BLAST2GO software v1.3.3 (https://www.blast2go.com/, Conesa et al., 2005) was used to reduce the data to the GOslim level (goslim_plant.obo) and perform basic statistics on ontological annotations as previously reported (Galla et al., 2009).

Annotations for genes involved in plant reproduction were retrieved from Galla et al. (2015). Additionally, a gene ontology-based annotation of the *Hypericum* flower transcriptome was attempted by querying the Amigo2 database (http://amigo.geneontology.org/) with opportune key words (such as “egg cell,” “central cell,” “synergic,” “antipodal cells,” “embryo sac,” “gamete,” “endosperm,” “meiotic,” and “cell fate”). In a parallel approach, the Amigo2 repository was queried for genes annotated as responsive to hormonal stimuli using the following search criteria: “response to abscisic acid,” “response to auxin,” “response to brassinosteroid,” “response to cytokinin,” “response to ethylene,” “response to gibberellin,” and “response to jasmonic acid.”

### Expression correlation network

An expression correlation network (Pearson correlation coefficient −0.98 <> +0.98) was created from all differentially expressed unigenes (fold change ≥ 2.0) using the plugin ExpressionCorrelation (http://apps.cytoscape.org/apps/expressioncorrelation) implemented in Cytoscape 3.4.0 (http://www.cytoscape.org/).

Sub-networks were generated from the overall expression correlation network (Pearson correlation coefficient −0.98 <> +0.98; 714 nodes, 8367 edges) by selecting nodes (DEGs) according to their annotations. Next, all adjacent edges and nodes connected to these edges were selected and used to infer the sub-network. For each sub-network, GO enrichment analyses were performed with the plugin BinGO with default parameters (*p* < 0.05, Benjamini & Hochberg's FDR correction with significance level 0.05).

### Validation of sequencing data by quantitative real-time PCRs

Plant materials were selected according to the genetic and cyto-histological bases of apospory recently described for *H. perforatum* (Schallau et al., 2010; Galla et al., 2011). Samples were collected separately from a minimum of five plant accessions (Table 1). RNA extractions were performed using a Spectrum™ Plant Total RNA Kit (Sigma-Aldrich). cDNA synthesis was performed using the RevertAid First Strand cDNA Synthesis Kit (Thermo Scientific), following the instructions of the supplier. Primers used in the Real-Time RT-PCR experiments are reported in Table S6. Expression analyses were performed using StepOne thermal cyclers and the 7300 Real-Time PCR System (Applied Biosystems), equipped with 96- and 384-well plate systems, respectively, with SYBR green PCR Master Mix reagent (Applied Biosystems). The amplification efficiency was calculated from raw data using OneStep Analysis software (Life Technologies). Amplification performance expressed as fold change was calculated with the ΔΔCt method using *HpTIP4* as a housekeeping gene (Pfaffl, 2001). Error bars indicate the standard error observed among the five biological replicates.

### Data availability

Raw sequences files were made available for download from SRA with the following accession numbers: SRR1646951, SRR1646953, SRR1646955, SRR1646956, SRR1647632, SRR1647633, SRR1647673, SRR1647674, SRR1647677, SRR1647678, SRR1647713, and SRR1647714. Sequences of unigenes investigated by Real-Time qPCR along with unigenes aligned to the BAC sequence HM061166.1 were deposited under the Transcriptome Shotgun Assembly project at DDBJ/EMBL/GenBank under the accession GBXG00000000. The version described in this paper is the second version, GBXG02000000.

The expression data discussed in this publication have been deposited in NCBI's Gene Expression Omnibus (Edgar et al., 2002) and are accessible through GEO Series accession number GSE84768 (https://www.ncbi.nlm.nih.gov/geo/query/acc.cgi?acc=GSE84768).

## Results

A transcriptome analysis was performed on pistils collected from sexual and aposporous genotypes (Table 1) at flower developmental stages spanning female sporogenesis and gametogenesis, i.e., floral stages 11–14 according to Galla et al. (2011). In sexual ovules, these stages span a number of critical developmental steps: the meiotic division of the megaspore mother cell, the degeneration of three reduced megaspores and the selection of a functional megaspore, and three successive mitotic divisions giving rise to a functional eight-nucleate embryo sac with an egg cell apparatus (Galla et al., 2011). Conversely, aposporous ovules are frequently characterized by the failure of the meiotic division followed by developmental program of one or multiple unreduced embryo sacs from aposporous initials differentiating from somatic cells of the nucellus (Galla et al., 2011).

### Expression analysis of sexual and apomictic *H. perforatum* pistils reveals highly differentiated transcriptome signatures at early stages of ovule development

**Figure 1:**
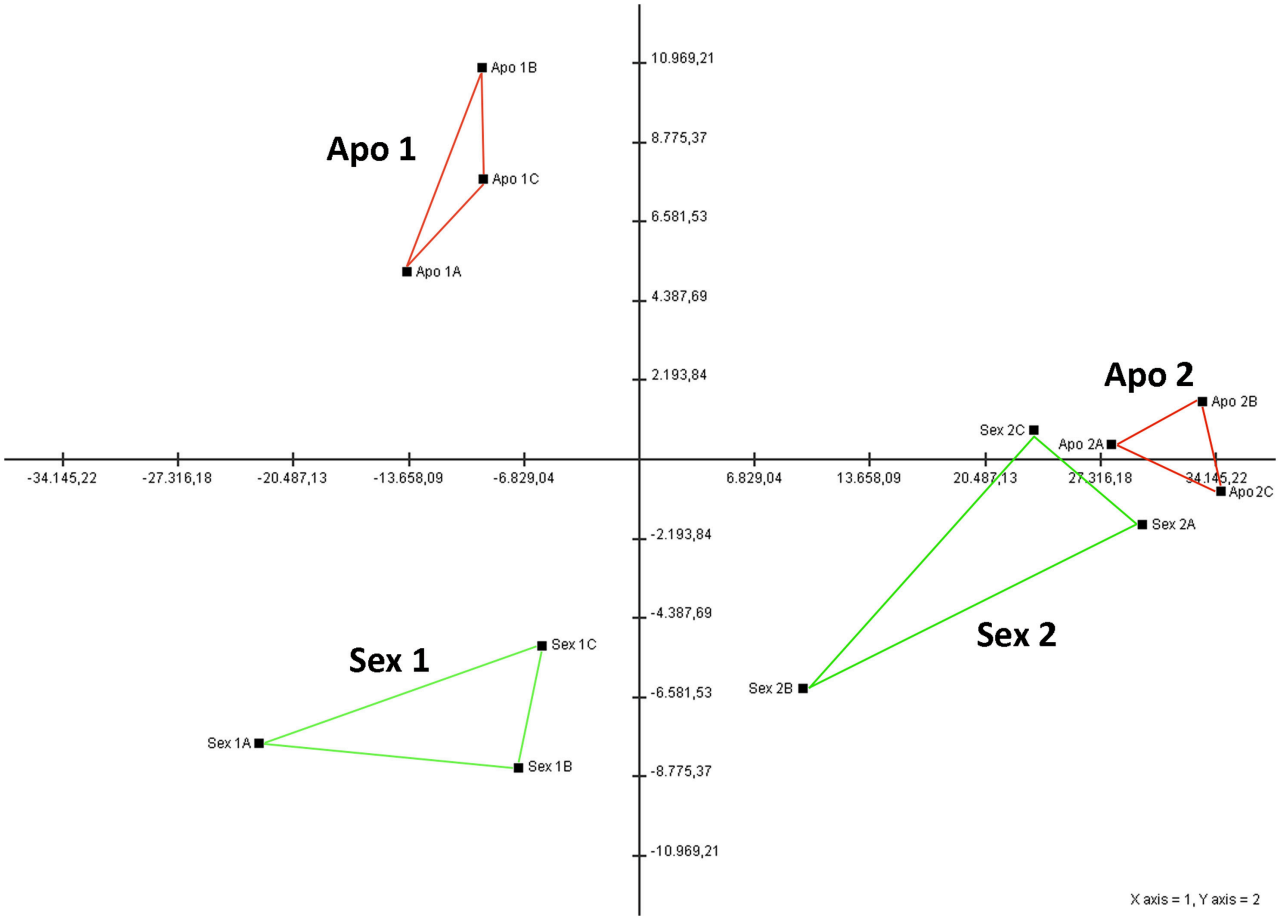
**Principal component analysis (PCA) of reproduction-related DEGs gene expression in sexual and aposporous pistils**. Sex1A-C, sexual samples at flower developmental stage 11; Sex2A-C, sexual samples at flower developmental stage 14. Apo1-C, apomictic samples at flower developmental stage 11; Apo2A-C, apomictic samples at flower developmental stage 14. Coloured lines were used to group the biological replicates. The percentage variation explained by the two axes is about 96%.

A principal component analysis (PCA) computed by using DEGs transcriptomic data allowed us to separate the sexual samples from the apomictic samples, with the two first principal components explaining approximately 51.5% of the overall variance (Figure S1). This analysis revealed that samples at flower developmental stages varying from meiosis (Sex1A, Sex1B, Sex1C) to gametogenesis (Sex2A, Sex2B, Sex2C) were closely related for the sexual genotypes whereas the apomictic genotypes revealed higher level of variation among samples of both early and late developmental stages (Figure S1). When considering only the expression data related to DEGs annotated as reproductive-related, centroids of the sexual and apomictic samples were plotted and clustered according to a different pattern, with the two first principal components explaining approximately 95% of the overall variance (Figure 1). This analysis revealed very high uniformity of biological replicates for the apomictic samples at both early and late developmental stages, whereas the sexual samples were characterized by higher variability among replicates, especially at the late developmental stages (Figure 1). In particular, apomictic samples collected at the late developmental stages, corresponding to gametogenesis, could be clearly separated from the apomictic samples collected at the early developmental stages, spanning apomeiosis and the differentiation of the aposporous initials. Altogether, the separation among sexual and apomictic samples collected at developmental stages spanning meiosis was very well marked, whereas sexual and apomictic samples collected during gametogenesis were closely related, indicating a lower level of variation in this stage (Figure 1).

Overall, across the two developmental stages, 224 and 973 unigenes were found to be significantly up- and down-regulated with a fold change ≥ 2 in at least one comparison, respectively. For convenience, clusters of unigenes that were up- and down-regulated in each comparison were designated C1-C8 (Table 2). A differential expression analysis was performed to identify genes modulated throughout pistil development in both sexual and apomictic genotypes (Table 2, clusters C1–C4), while at the same time providing the opportunity to compare sexual and apomictic transcriptomes at comparable pistil developmental stages spanning meiosis, apomeiosis and gametogenesis (Table 2, clusters C5–C8).

In total, 33 and 137 unigenes were up-regulated between the two selected developmental stages of pistils, in sexual (Table 2, cluster C1) and apomictic (Table 2, cluster C3) genotypes, respectively. Conversely, the numbers of down-regulated unigenes in pistils collected at the same developmental stages were 53 and 182, respectively, for sexual (Table 2, cluster C2) and apomictic (Table 2, cluster C4) genotypes.

The comparison between sexual and apomictic pistils spanning early flower developmental stages, which are characterized by the circumvention of the meiotic program and the onset of cellular and biological modifications leading to the differentiation of aposporous initials, underlined as many as 760 unigenes that were differentially expressed (Table 2, clusters C5 and C6). This comparison provided the highest number of DEGs. We also noticed a marked imbalance in the number of DEGs between sexual and apomictic pistils at early flower developmental stages, with 36 up- and 724 down-regulated, respectively (Table 2, clusters C5 and C6). Interestingly, the comparison between sexual and apomictic pistils collected at late flower developmental stages (floral stage 14, clusters C7 and C8) was characterized by 32 DEGs, 18 and 14 of which were up- and down-regulated, respectively (Table 2, cluster C8).

Differences in the expression of transcripts encoded by genes located on the BAC clone encompassing the genomic locus associated with apospory (Schallau et al., 2010) were not significant (considering a fold change >2) in the pair-wise comparisons between reproductive strategies and developmental stages examined in this study (Figure S2). However, the unigene HHKO36P02G7QAY, mapping on the BAC region encoding for the protein HnRNP, was significantly differentially expressed between sexual and apomictic pistils at floral stage 11 (fold change is equal to-1.89).

**Figure 2:**
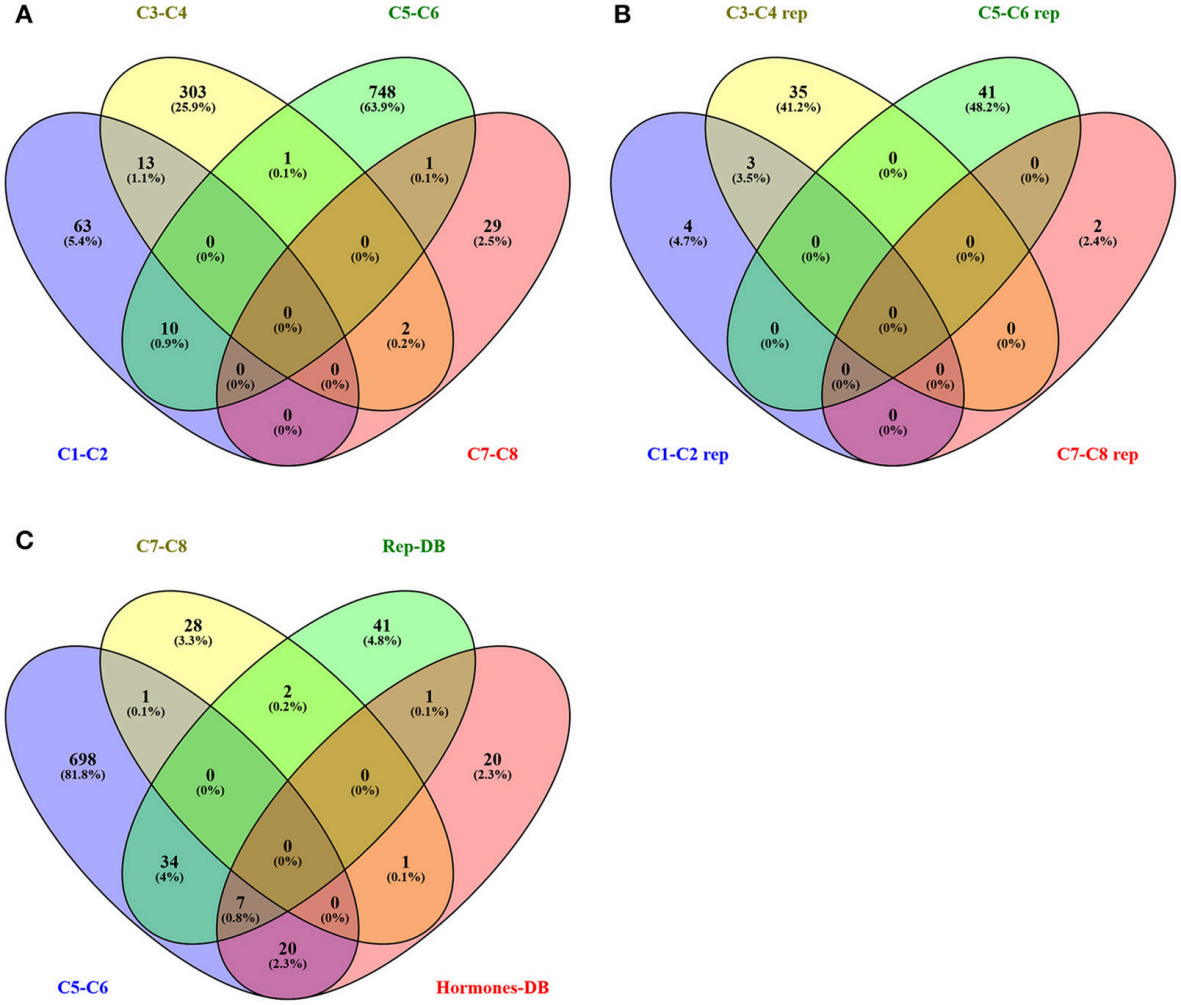
**Venn diagrams graphically represent the number of unigenes differentially expressed in single or multiple comparisons. (A)** Number of DEGs identified in clusters C1-C8. **(B)** Number of reproduction-related DEGs identified in clusters C1-C8. **(C)** DEGs identified in clusters C5-C6 and C7-C8, DEGS annotated as reproduction-related (Rep-DB) and DEGs annotated as responsive to hormonal stimuli (Hormones-DB).

Venn diagrams were calculated to estimate the overlaps between unigenes differentially expressed in apomictic samples compared to the sexual samples and unigenes which, based on annotations of their homologs, are likely associated with sporogenesis and/or gametogenesis. In doing so, we discovered that 13 unigenes belonging to the clusters C1 and C2 were also included in clusters C3 and C4 (Figure 2A). Likewise, 10 unigenes belonging to clusters C1 or C2 were also included in the clusters C5 and C6. Notably, only the unigene isotig20049, sharing high similarity with the *A. thaliana* ribosome maturation protein SBDS encoded by the gene AT1G43860, appeared to be upregulated in apomictic samples across the early and late developmental stages (clusters C5/C7).

The ontological annotation of DEGs was performed according to two vocabularies: biological processes and molecular functions. Of the eight datasets, the numbers of differentially expressed genes that were annotated with at least one GO term were 63 (0.73% of clusters C1/C2), 265 (0.83% of clusters C3/C4), 474 (0.62% of clusters C5/C6) and 17 (0.53% of clusters C7/C8). For biological processes, the highest scores were recorded for terms associated with the sensing and response to stresses and stimuli as well as for terms associated with the metabolism of carbohydrates, lipids and secondary compounds (Table 3). The annotations of anatomical structure morphogenesis (hits: 47, GO:0009653), cellular component organization (hits: 116, GO:0016043), cell cycle (hits: 71, GO:0007049), DNA metabolic process (hits: 80, GO:0006259) and signal transduction (hits: 45, GO:0007165) were also identified to a relatively high extent (Table 3).

**Table 3:** **Annotation of differentially expressed unigenes according to the GO vocabulary: biological process**.

**GO-ID**	**GO-term**	**SexS11 /SexS14**	**ApoS11 /ApoS14**	**SexS11 /ApoS11**	**SexS14 /ApoS14**
GO:0000003	reproduction	6	0	0	1
GO:0005975	carbohydrate metabolic process	0	14	25	1
GO:0006091	generation of precursor metabolites and energy	0	0	5	0
GO:0006259	DNA metabolic process	8	40	30	2
GO:0006412	translation	0	0	9	0
GO:0006464	cellular protein modification process	0	29	34	2
GO:0006629	lipid metabolic process	0	10	18	1
GO:0006810	transport	7	21	60	3
GO:0006950	response to stress	6	41	62	3
GO:0007049	cell cycle	5	43	21	2
GO:0007165	signal transduction	0	17	26	2
GO:0009056	catabolic process	0	9	29	1
GO:0009058	biosynthetic process	15	74	0	5
GO:0009605	response to external stimulus	0	13	0	2
GO:0009606	tropism	0	0	5	0
GO:0009607	response to biotic stimulus	0	10	16	1
GO:0009628	response to abiotic stimulus	5	25	43	2
GO:0009653	anatomical structure morphogenesis	6	22	19	0
GO:0009719	response to endogenous stimulus	0	15	26	1
GO:0009790	embryo development	0	9	13	0
GO:0009791	post-embryonic development	8	0	0	1
GO:0009856	pollination	0	0	6	0
GO:0009908	flower development	0	9	19	0
GO:0009991	response to extracellular stimulus	0	0	6	0
GO:0015979	photosynthesis	0	0	0	1
GO:0016043	cellular component organization	8	56	48	4
GO:0016049	cell growth	0	8	7	0
GO:0019725	cellular homeostasis	0	0	6	0
GO:0019748	secondary metabolic process	0	0	5	0
GO:0030154	cell differentiation	0	14	11	0
GO:0040007	growth	0	0	0	1
GO:0040029	regulation of gene expression. epigenetic	0	7	7	0

*For each GO-ID the table reports on the GO-term and number of annotated unigenes identified in each comparison*.

Differentially Expressed Genes (DEGs) annotated as reproduction were only included in clusters C1/C2 (hits: 6) and C7/C8 (hits: 1), while pollination was only present in clusters C5/C6 (hits: 6). Additional GO terms assigned to DEGs included in the clusters C5/C6 were flower development (hits: 9), embryo development (hits: 9), cell growth (hits: 8) and cell differentiation (hits: 14). It is worthy of mention that these latter GO terms were also associated with DEGs belonging to the clusters C3/C4 (Table 3).

The annotation of DEGs according to the putative molecular functions of corresponding gene products revealed a number of terms assigned with higher frequency (Table 4). In this case, the highest scores were recorded for GO terms associated with nucleotide binding (GO:0000166), DNA binding (GO:0003677), transporter activity (GO:0005215) and protein binding (GO:0005515). Overall, 54 DEGs were annotated as having sequence-specific DNA binding transcription factor activity (GO:0003700). Among DEGs belonging to the clusters C5/C6, which are thus likely reflective of transcriptional changes associated with apomeiosis and cell fate changes needed for apospory initiation, several unigenes were annotated as chromatin binding (hits: 8, GO:0003682), RNA binding (hits: 27, GO:0003723) and nuclease activity (hits: 15, GO:0004518). A GO enrichment analysis allowed the identification of GO terms significantly over-represented in each cluster of DEGs (*p* < 0.05, FDR < 0.05). While clusters 2, 7, and 8 were not enriched for any ontological terms (Table S1), DEGs included in clusters C5 and C6 were enriched for multiple terms, including cell cycle (GO:0007049, FDR: 2.39E-23), DNA metabolic process (GO:0006259, FDR: 2.70E-23), single-organism cellular process and related terms (GO:0044763, FDR: 5.76E-12) (Table S1). For molecular functions, the highest scores were recorded for terms associated with DNA binding (GO:0003677, FDR: 1.31E-10), DNA (cytosine-5-)-methyltransferase activity (GO:0003886, FDR: 3.52E-08) and heterocyclic compound binding (GO:1901363, FDR: 8.46E-07), among others (Table S1). Notably, despite the abundance of unigenes included in clusters C5 and C6, very few ontological terms appeared to be significantly enriched for annotations associated with the included unigenes. Thus, transcription corepressor activity (GO:0003714, FDR: 2.84E-02) and regulation of jasmonic acid mediated signaling pathway (GO:2000022, FDR: 2. 84E-02) were the only enriched ontological terms in cluster 5, which included 36 unigenes. No ontological annotations related to molecular functions and biological processes were found to be significant in unigenes included in cluster C6. Summarizing, the ontological annotation of DEGs provided clear indications concerning the biological process and molecular functions that are overrepresented in concomitance to the early events of the aposporous developmental pathway.

**Table 4:** **Annotation of differentially expressed unigenes according to the GO vocabulary: molecular function**.

**GO-ID**	**GO-term**	**SexS11 /SexS14**	**ApoS11 /ApoS14**	**SexS11 /ApoS11**	**SexS14 /ApoS14**
GO:0000166	nucleotide binding	6	46	69	4
GO:0003677	DNA binding	9	52	42	2
GO:0003682	chromatin binding	0	12	8	1
GO:0003700	sequence-specific DNA binding transcription factor activity	7	22	23	2
GO:0003723	RNA binding	0	0	27	1
GO:0003774	motor activity	0	10	0	0
GO:0004518	nuclease activity	0	0	15	0
GO:0004871	signal transducer activity	0	0	7	1
GO:0004872	receptor activity	0	0	5	1
GO:0005198	structural molecule activity	0	0	9	0
GO:0005215	transporter activity	5	14	39	2
GO:0005515	protein binding	13	57	60	6
GO:0008289	lipid binding	0	0	9	1
GO:0016301	kinase activity	0	30	32	0
GO:0016787	hydrolase activity	16	0	0	2
GO:0019825	oxygen binding	0	0	6	0
GO:0030234	enzyme regulator activity	0	6	5	0
GO:0030246	carbohydrate binding	0	0	5	0

*For each GO-ID the table reports on the GO-term and number of annotated unigenes identified in each comparison*.

Real-Time RT-qPCR assays were used to corroborate the microarray expression data for a number of key genes, which were selected on the bases of their expression pattern or annotation. Nine genes were selected among those differentially expressed at early developmental stages in apomictic vs. sexual samples (Figure S3). The piezo-type mechanosensitive ion channel encoded by the unigene isotig06122 exhibited a significant reduction in its expression in apomictic samples at floral stage 11, while no significant difference in its expression was recorded at floral stage 14. The same expression pattern was observed for the unigene isotig06679, orthologous to the *Arabidopsis* gene IDN2, which was found to be down regulated in apomictic pistils collected at developmental stages encompassing apomeiosis and induction of the aposporous developmental program (i.e., floral stage 11). The expression of unigene isotig06027 (similar to AT5G04390) decreased in apomictic samples in correspondence of both developmental stages. A decreased expression level in aposporous pistils was also observed for the three unigenes: isotig23509 (similar to the C2H2-type zinc finger protein encoded by AT5G04390), isotig16989 (similar to the mithocondrial translocase encoded by AT5G43970) and isotig07554 (similar to the the alpha-expansin 11 encoded by At1g20190) at floral stage 14 (Figure S3). The two unigenes: isotig12158 and isotig17364, encoding for a MYB7- like transcription factor and an ankyrin repeat family protein, showed the same expression pattern in sexual and apomictic samples and appeared to be modulated were to be modulated during pistil development. The unigene isotig17171, encoding for a protein of unknown function, was up-regulated in apomictic pistils in correspondence of both developmental stages. (Figure S3).

### Phenotypic expression of apospory is concomitant with the modulation of key genes involved in the sexual reproductive pathway and the response to hormonal stimuli

We investigated the hypothesis of deregulation of the sexual developmental pathway leading to apomixis (Grimanelli et al., 2001) by identifying DEGs associated with reproduction based on an annotated *H. perforatum* flower transcriptome (Galla et al., 2015). In doing so, we could annotate 2512 unigenes matching 1042 *Arabidopsis* gene models as related to plant reproduction, which include a number of DEGs (Table S2).

Six unigenes matching five *Arabidopsis* gene models were annotated as sporophytic mutants with gametophytic defects (Bencivenga et al., 2012). Despite the fact that gene products are mostly expressed in *Arabidopsis* sporophytic tissues, these genes are known to lead to gametophytic defects when mutated (Table S3). Additionally, 34 and 25 unigenes were annotated as sporogenesis- and gametogenesis-related, respectively (Table S3). Remarkably, the vast majority of these unigenes was included in clusters C4 and C6 and was down-regulated in aposporous pistils at floral stage 11.

Based on the expression patterns of these genes in sexual and apomictic pistils, 7 and 38 reproductive-related DEGs were included in clusters C1/C2 and C3/C4, respectively (Figure 2B, Table S3). Furthermore, 43 unigenes that, based on the annotation of their *Arabidopsis* homologs were annotated with GO terms linked to cell fate commitment, sporogenesis, gametogenesis or embryogenesis, were differentially expressed (*p* < 0.01, FC ≥ 2.0) in the C5-C8 comparison (Table S3). Of these 38 were down-regulated in pistils of aposporous genotypes (C6/C8), whereas five were significantly up-regulated at both floral developmental stages 11 and 14 (C5/C7).

Next, in the attempt to identify additional levels of regulation acting during sporogenesis and gametogenesis, the *H. perforatum* flower transcriptome was annotated with terms for hormonal regulation, whereby annotations were provided by TAIR. Within the flower transcriptome, 2436 unigenes were annotated as responsive to hormonal stimuli (Table S2).

Further categorization of DEGs based on their commitment to hormonal biosynthesis, metabolism and signaling pathways is reported in Table 5. Among these genes, a restricted set of DEGs were involved in cytokinin biosynthesis, such as the tRNA dimethylallyltransferase 2 HPIPT2, and auxin homeostasis, such as HPPIN8, HPIAR3 and HPILL6. Similarly, we identified a number of genes that, based on their annotations, could be recognized as regulators of hormonal stimuli (Table 5). Remarkably, the two cytokinin receptor-like proteins HPCRE1 and HPACR4 were down-regulated in aposporous pistils at floral stage 11 (cluster C6, Table 5). Multiple auxin responsive factors (HPARF2, HPARF3, HPARF7) and SAUR-like proteins were also included in clusters C1-C8 (Table 5).

**Table 5:** **Differentially expressed genes whose expression is predicted to be modulated in response to hormonal stimuli**.

**Annotation**	**Unigene ID**	**AT top hit**	**Gene names**	**Protein names**	**Reproductive database**	**Cluster (FC)**
cytokinin biosynthetic process	isotig31470	AT2G27760	IPT2	tRNA dimethylallyltransferase 2	meiotic	C6 (−2.23)
cytokinin signaling pathway	F6Z56EY03HBNM2	AT2G01830	AHK4/CRE1/ WOL	Histidine kinase 4	-	C6 (−2.17)
response to cytokinin	F43FOUR02DAHH0	AT1G69040	ACR4	ACT domain-containing protein ACR4	sporophytic mutants with gametophytic defects	C6 (−2.28)
response to cytokinin	HHKO36P01AWLS2	AT3G54720	AMP1	Probable glutamate carboxypeptidase 2	embryogenesis	C6 (−2.58)
response to cytokinin	isotig30471	AT3G54720	AMP1	Probable glutamate carboxypeptidase 2	embryogenesis	C6 (−2.22)
response to cytokinin	isotig01327	AT4G02450	AT4G02450	HSP20-like chaperone	-	C6 (−2.38)
response to cytokinin	isotig13937	AT4G02530	AT4G02530	Thylakoid lumenal 16.5 kDa protein	-	C4 (−2.56)
response to cytokinin	isotig00876	AT5G50920	CLPC1	Chaperone protein ClpC1	-	C6 (−2.17)
response to cytokinin	isotig08889	AT5G35630	GS2	Glutamine synthetase	-	C4 (−6.05)
response to cytokinin	isotig09922	AT1G32060	PRK	Phosphoribulokinase	-	C4 (−2.54)
response to auxin	isotig18540	AT1G51760	ILL4/IAR3/JR3	IAA-amino acid hydrolase ILR1-like 4	-	C3 (2.42)
auxin transport	isotig28540	AT5G16530	PIN8	Putative auxin efflux carrier component 8	-	C6 (−2.09)
basipetal auxin transport	isotig08210	AT4G30960	CIPK6/PKS4/SIP3	CBL-interacting serine/threonine-protein kinase 6	-	C3 (5.47)
basipetal auxin transport	isotig08211	AT4G30960	CIPK6/PKS4/SIP3	CBL-interacting serine/threonine-protein kinase 6	-	C3 (5.85)
auxin signaling pathway	isotig05394	AT5G62000	ARF2	auxin response factor 2	seed development and size regulation	C6 (−2.17)
auxin signaling pathway	isotig22461	AT2G33860	ARF3/ETT	auxin response transcription factor 3	cell fate	C4 (−2.05)
auxin signaling pathway	isotig02453	AT5G20730	ARF7/NPH4	Auxin response factor	-	C6 (−3.29)
auxin signaling pathway	isotig13218	AT5G43700	ATAUX2-11/IAA4	auxin-responsive protein IAA4	-	C3 (6.04)
auxin signaling pathway	isotig01915	AT5G18020	SAUR20	Auxin-responsive protein SAUR20	-	C3 (2.29)
auxin signaling pathway	isotig05167	AT5G46700	TRN2/TET1	protein TORNADO 2	regulation of megasporogenesis	C6 (−2.13)
auxin/ethylene signaling pathway	isotig32883	AT5G42190	SKP1B/ASK2/UIP2	SKP1-like protein 1B	-	C6 (−2.58)
response to auxin	isotig15559	AT5G20820	AT5G20820	SAUR-like auxin-responsive family protein	-	C6 (−2.07)
response to auxin	F6Z56EY02ECT02	AT1G19840	AT1G19840	SAUR-like auxin-responsive protein	-	C2 (−2.33)
response to auxin	isotig16626	AT2G04160	AIR3/SBT5.3	subtilisin-like serine endopeptidase family protein	-	C1 (6.34)
response to auxin	isotig16625	AT2G04160	AIR3/SBT5.3	subtilisin-like serine endopeptidase family protein	-	C1 (6.27)
response to auxin	isotig23915	AT3G55320	ABCB20/MDR14	ABC transporter B family member 20	-	C6 (−2.47)
response to auxin	isotig04082	AT5G19140	AILP1	aluminum induced protein with YGL and LRDR motifs	-	C5 (2.58)
response to auxin	HHKO36P01B6PR4	AT5G01270	CPL2	RNA polymerase II C-terminal domain phosphatase-like 2	-	C6 (−3.19)
brassinosteroid signaling pathway	isotig24963	AT3G63310	F16M2_160/BIL4	BRZ-INSENSITIVE-LONG HYPOCOTYLS 4	-	C3 (5.01)
brassinosteroid signaling pathway	isotig05518	AT4G39400	BRI1	Serine/threonine-protein kinase bri1	regulation of megagametogenesis	C6 (−4.10)
ethylene signaling pathway	HHKO36P01EDKCF	AT1G36060	ERF055	Ethylene-responsive transcription factor	-	C6 (−2.23)
ethylene/abscisic acid/brassinosteroid signaling pathway	isotig03782	AT3G51550	FER/ SIR/SRN	Receptor-like protein kinase	pollen tube reception	C6 (-2.08)
response to ethylene; gibberellic acid signaling pathway	isotig24822	AT2G01570	RGA/GRS/RGA1	GRAS family transcription factor	-	C3 (4.19)
response to ethylene	isotig31421	AT3G54320	WRI1	Ethylene-responsive transcription factor	-	C3 (6.47)
cellular response to abscisic acid stimulus	isotig24487	AT1G10930	RECQ4A/SGS1	ATP-dependent DNA helicase Q-like 4A	-	C4 (−2,92)
abscisic acid signaling pathway	isotig23433	AT2G31500	CPK24	Calcium-dependent protein kinase, putative	-	C3 (2.16)
response to abscisic acid	HHKO36P01B0WID	AT4G13510	AMT1-1	Ammonium transporter 1 member 1	-	C6 (−3.53)
response to abscisic acid	isotig18052	AT2G36530	LOS2/ENO2	Bifunctional enolase 2/transcriptional activator	-	C5 (2.67)
response to abscisic acid	isotig02231	AT5G59310	LTP4	Non-specific lipid-transfer protein 4	-	C6 (−3.00)
response to abscisic acid	isotig02230	AT5G59310	LTP4	Non-specific lipid-transfer protein 4	-	C6 (−3.12)
response to abscisic acid	isotig16604	AT1G27730	STZ/ZAT10	Zinc finger protein ZAT10	-	C7 (2.40)
response to abscisic acid	isotig20742	AT3G07360	PUB9	U-box domain-containing protein 9	-	C3 (3.47)
response to abscisic acid	isotig27335	AT5G25610	RD22	BURP domain protein RD22	-	C4 (−2.29)
jasmonic acid biosynthetic process	isotig19051	AT2G33150	PED1/KAT2	3-ketoacyl-CoA thiolase 2	-	C3 (2.30)
jasmonic acid signaling pathway	isotig02241	AT5G20900	TIFY3B/JAZ12	Jasmonate ZIM domain-containing protein 12		C5 (2.04)
jasmonic acid signaling pathway	isotig02239	AT5G20900	TIFY3B/JAZ12	Jasmonate ZIM domain-containing protein 12		C5 (2.04)
response to jasmonic acid	isotig06160	AT1G19180	JAZ1	Jasmonate ZIM-domain containing protein	regulation of megagametogenesis	C5 (2.23)
response to jasmonic acid	isotig24727	AT1G44350	ILL6/GR1	IAA-amino acid hydrolase	-	C3 (10.27)
response to jasmonic acid	isotig09757	AT4G12570	UPL5	E3 ubiquitin-protein ligase UPL5		C4 (−2.93)
gibberellic acid signaling pathway	isotig23198	AT5G66730	IDD1/ENY	Protein indeterminate-domain 1		C6 (−2.26)
gibberellic acid/brassinosteroid signaling pathway	isotig26633	AT5G39860	PRE1/BHLH136/BNQ1	Transcription factor PRE1	-	C1 (2.80)
response to gibberellin/jasmonic acid/salicylic acid	isotig13103	AT4G09460	MYB6	Transcription repressor MYB6	-	C6 (−3.96)
response to gibberellin	F43FOUR04I6R08	AT4G19700	BOI/ILP	E3 ubiquitin-protein ligase BOI	-	C6 (−2.89)
hormone-signaling pathway	isotig07074	AT1G20930	CDKB2-2	Cyclin-dependent kinase B2-2	-	C4 (−5.14)

*For each unigene the table reports on its involvement in hormonal biosynthesis and/or perception. The Arabidopsis gene sharing the highest similarity, the gene name, the protein name, annotations related to reproduction and differential expression (gene expression cluster and fold change) are also indicated*.

The overlaps existing between DEGs differentially expressed in aposporous vs. apomictic samples (clusters C5–C8), those annotated as reproduction-related (namely, “reproductive-related”) and unigenes thought to be responsive to hormonal stimuli (namely, “response to hormonal stimuli”) are displayed in Figure 2C. While the majority of DEGs were not annotated according to the abovementioned categories, 7 DEGs matching 6 *A. thaliana* gene models were included in both datasets (Table 5, Figure 2C). These unigenes were annotated as sporophytic mutants with gametophytic defects (HPACR4) and regulators of megasporogenesis (HPTRN2) as well as regulators of megagametogenesis progression and gametophyte development (HPBRI1, HPJAZ1). The *Hypericum* homologs of HPFER and HPARF2, the former of which is involved in pollen tube perception and the latter of which is involved in gametophyte and seed development in *A. thaliana*, were also among these genes (Table 5).

Real-Time qPCR assays showed that the unigene HPACR4 (F43FOUR02DAHH0), encoding for a protein involved in epidermal cell differentiation, exhibited a significant reduction in its expression in apomictic samples in correspondence of both floral stages (Figure S4). The unigene isotig28540, orthologous to the *Arabidopsis* gene PIN8, exhibited an opposite expression pattern as it was significantly upregulated in apomictic pistils in correspondence of both floral stages (Figure S4). Conversely, no significant differences were recorded by Real-Time RT-qPCR assays for HPIPT2 (isotig31470), HPCRE1 (F6Z56EY03HBNM2) and HPAMP1 (HHKO36P01AWLS2) (Table S7, Figure S4). Summarising, our expression analysis provides line of evidences suggesting that early events of the aposporous developmental pathway are concomitant with the modulation of multiple genes involved in the sexual reproductive pathway and hormonal homeostasis.

### The transcriptome of sexual and aposporous pistils is characterized by distinct expression correlation networks

An expression correlation network (Pearson correlation coefficient −0.98 <> +0.98) was created starting from all differentially expressed unigenes (fold change ≥ 2.0). Sub-networks were generated from the original network by selecting nodes (i.e., unigenes) modulated in single comparisons, whereby highly correlated unigenes were connected to each other (Table S4).

The expression correlation network originated by selecting unigenes included in clusters C1 and C2, thus showing differential expression during the transition from early to late developmental stages in sexual genotypes, comprised 71 nodes interconnected by 63 edges. Based on their annotations, 11 unigenes included in this network are likely involved in meiosis (i.e., the kinesin-related cytokinesis protein F2H17_19), as well as ovule development and gamete formation (i.e., HPANT). Furthermore, 5 unigenes were annotated as responsive to auxin (HPAIR3), ethylene (HPASK2) and Brassinosteroid (HPBRI1) stimuli. Following a GO enrichment analysis, ontological terms describing DNA metabolic processes (GO:0006259, *p*-value: 1.23E-5), DNA replication (GO:0006260, *p*-value: 1.66E-4), DNA-methyltransferase activity (GI:0009008, *p*-value: 3.48E-4) and gene silencing (GO:0016458, *p*-value: 2.52E-4) were detected starting from this cluster of DEGs (Table S5). Biological processes significantly over-represented in the annotations of nodes included in this network were zygote asymmetric cell division (GI:0010070, *p*-value: 1.81E-4), regulation of flower development (GI:0009909, *p*-value: 5.09E-3) and mitotic chromosome condensation (GI:0007076, *p*-value: 5.31E-3), among others (Table S5).

The network originated by selecting DEGs included in clusters C3 and C4 comprised 154 nodes and 247 edges. Within this network, 5 unigenes are known to be modulated in response to ethylene (WRI1), abscisic acid (PKT3), jasmonate (UPL5, ILR) and other hormonal stimuli (CDKB2;2). Furthermore, 18 DEGs were annotated as reproduction-related genes with terms ascribable to meiosis (BUB3.1, PHB, TOP2), gametogenesis (TSO1, T1N15_20) and cell fate changes (RBR1, HMGA). The *Hypericum* orthologues of MET1 and CMT3 were also included in this cluster of modulated genes. Within this sub-network, terms scoring the highest values were associated with cell cycle progression (GO:0022402, *p*-value: 2.40E-14), DNA replication (GO:0006260, *p*-value: 1.35E-7) and DNA metabolic process (GO:0006259, *p*-value: 1.77E-7), meiosis (GO:0007126, *p*-value: 6.9E-4) and chromosome segregation (GO:0007059, *p*-value: 1.30E-4) (Table S5). Several terms related to cell fate commitment (GO:0045165, *p*-value: 7,27E-03) were also included among over-represented terms. It is worth noting that the terms basipetal auxin transport (GO:0010540, *p*-value: 7,27E-03) and IAA-amino acid conjugate hydrolase activity (GO:0010178, *p*-value: 5,22E-03) were significantly enriched in annotations assigned to this cluster of genes (Table S5).

**Figure 3:**
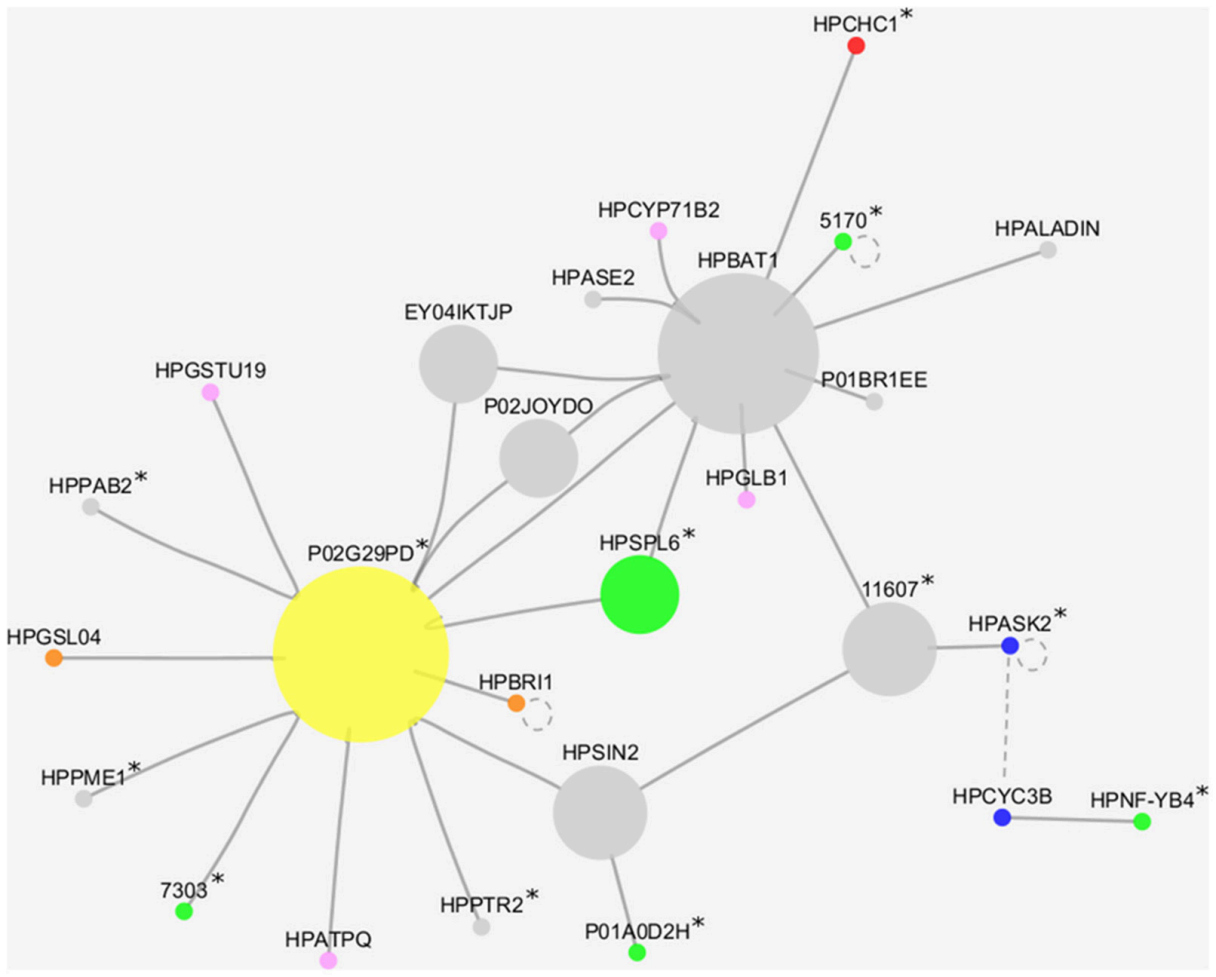
**Cytoscape visualization of the expression correlation sub-network generated by filtering all DEGs as: [(C1 OR C2) AND (C5 OR C6)]**. Nodes size is proportional to number of connections. Green nodes, transcription factors; blue nodes, cell cycle regulators; red node, chromatin remodeling factor; orange nodes, reproductive-related unigenes; yellow node, transposable elements; pink nodes, stress related proteins; gray nodes, other annotations. Continuous lines indicate edges identified in the expression correlation network while dotted lines indicate known physical interactions between the *A. thaliana* orthologues. Unigenes whose expression has been validated by Real-Time qPCR are indicated with the asterisk (^*^).

An expression correlation network (Pearson correlation coefficient −0.98 <> +0.98) was created starting from unigenes (nodes) included in clusters C1, C2, C5, and C6, thus possibly reflecting a subset of expression changes concomitant to the failure of the meiotic program and the induction of aposporous apomixis. This approach was selected to scale down the complexity of data, thus allowing closer investigations of selected nodes. The resulting expression correlation network contains two sub-networks for a total of 26 nodes (unigenes) and 29 edges (Figure 3, Table 6). All edges included in the network represent positive correlations between unigenes that were found down-regulated in aposporous pistils. For these genes, fold changes varied between a minimum value of 2.01 for the unigene of unknown function HHKO36P01BR1EE and a maximum fold change value equal to 13.68 for the mitochondrial ribosome-associated GTPase 1 encoded by the unigene F6Z56EY02DHSUQ. In addition to being differentially expressed during early stages of pistil development between sexual and apomictic genotypes, five unigenes included in the network were significantly down-regulated during sexual development (cluster C2) (Table 6).

**Table 6:** **Unigenes included in the expression correlation sub-network potentially associated with the induction of the aposporous developmental program**.

**Unigene**	**AT locus**	**Gene name**	**Description**	**Biological processes**
isotig25064	AT5G14170	CHC1	Chromatin remodeling complex subunit	chromatin modification; regulation of transcription, DNA-templated
HHKO36P01A0D2H	AT3G62900	-	CW-type Zinc Finger-like protein	n.a.
isotig05170	AT1G55750	-	BSD domain (BTF2-like transcription factors)	nucleotide-excision repair; regulation of transcription, DNA-templated
isotig05639	AT1G69170	SPL6	Squamosa promoter-binding protein-like transcription factor	defense response to bacterium; regulation of transcription, DNA-templated
isotig07303	AT5G63830	-	HIT-type Zinc finger family protein	n.a.
isotig32883	AT5G42190	ASK2	Similar to ARABIDOPSIS SKP-LIKE 2	auxin-activated signaling pathway; ubiquitin-dependent protein catabolic process
F6Z56EY02DHSUQ	AT4G10650	SIN2	mitochondrial ribosome-associated GTPase 1	GTP-binding; hydrolase activity
isotig24694	AT4G34110	PAB2	Poly(A)-binding protein	nuclear-transcribed mRNA catabolic process, nonsense-mediated decay
isotig05518	AT4G39400	BRI1	Serine/threonine-protein kinase bri1	anther wall tapetum cell differentiation; negative regulation of cell death
isotig11607	AT5G41940	-	Ypt/Rab-GAP domain of gyp1p superfamily protein	intracellular protein transport; regulation of vesicle fusion
isotig13015	AT3G56900	ALADIN	Transducin/WD40 repeat-like superfamily protein	mRNA transport; protein transport
F6Z56EY04IKTJP	AT1G74150	-	kelch repeat superfamily protein	n.a.
isotig18376	AT2G01170	BAT1	Amino-acid permease.	arginine transmembrane transport
isotig27808	AT3G14570	GSL04	GLUCAN SYNTHASE-LIKE 4	(1->3)-beta-D-glucan biosynthetic process
isotig22811	AT1G78380	GSTU19	GLUTATHIONE S-TRANSFERASE TAU 19	glutathione metabolic process; response to oxidative stress
HHKO36P02G29PD	AT2G14380	-	putative retroelement pol polyprotein	n.a.
isotig06992	AT1G13080	CYP71B2	CYTOCHROME P450, FAMILY 71, SUBFAMILY B, POLYPEPTIDE 2	defense response to other organism; heat acclimation
isotig07093	AT2G02040	PTR2	Predicted protein	peptide transport; protein transport
isotig12044	AT1G53840	PME1	PECTIN METHYLESTERASE 1	cell wall modification; pectin catabolic process
HHKO36P01BR1EE	-	-	Unknown product	n.a.
HHKO36P01C9NWL	AT4G34740	ASE2	DIFFERENTIAL DEVELOPMENT OF VASCULAR ASSOCIATED CELLS	de novo IMP biosynthetic process; chloroplast organization
HHKO36P02G1N94	AT3G52300	ATPQ	ATP SYNTHASE D CHAIN, MITOCHONDRIAL	ATP synthesis coupled proton transport; response to salt stress
HHKO36P02GPA6L	AT2G16060	GLB1	CLASS I HEMOGLOBIN	response to hypoxia
HHKO36P02JOYDO	AT3G63510	-	FMN-linked oxidoreductases superfamily protein	flavin adenine dinucleotide binding; tRNA dihydrouridine synthase activity
F43FOUR03GUMNU	AT1G09030	NF-YB4	nuclear transcription factor Y subunit B-4-like	regulation of transcription, DNA-templated
HHKO36P02GL54X	AT5G11300	CYC3B	MITOTIC-LIKE CYCLIN 3B	cell cycle; cell division; cell proliferation

*To generate the sub-network from the overall expression correlation network (Pearson correlation coefficient −0.98<> +0.98), all nodes (DEGs) included in clusters (C1 OR C2) AND (C5 OR C6) were selected. Next, all adjacent edges and nodes connected to these edges were selected and used to infer the sub-network. For each unigene the table reports on the putative Arabidopsis orthologous, the gene name and the description of the gene. The relevant biological processes attributed to each unigene are also indicated*.

Based on their annotations, unigenes included in the network could be further classified into the following ontological categories: (i) transcriptional regulators, including SPL (SQUAMOSA promoter-binding protein-like transcription factor), BSD domain (BTF2-like transcription factors), the nuclear transcription factor Y subunit B-4-like and a chromatin remodeling complex subunit (CHC1); (ii) translation regulators, including the PAB2 Poly(A)-binding protein and a mitochondrial ribosome-associated GTPase 1; (iii) cell cycle regulators, such as SKP-like 2 and mitotic-like cyclin 3B; (iv) proteins involved in signal perception and transduction (the Serine/threonine-protein kinase (BRI1) was included in this group (Table 6); v) unigenes responsive to internal or external stresses and stimuli, such as glutathione S-transferase TAU 19 (GSTU19), cytochrome P450-like gene CYP71B and ATP synthase D chain (ATPQ); and (vi) genes encoding for proteins involved in cellular modification processes, such as the pectinesterase PME1 and glucan synthase-like 4 (GSL04). Based on annotations available at UniProt, this latter gene could also be included in the group of genes that are known to be involved in plant reproduction together with BRI1 (Table 6).

Remarkably, the nodes sharing the highest numbers of interactions within the sub-network were isotig18376 (HPBAT1) and HHKO36P02G29PD, this latter encoding for a putative retroelement pol polyprotein. We noticed that transcription factors included in this picture were localized in marginal positions. The only exception to this was represented by the SQUAMOSA promoter-binding protein-like transcription factor 6 (HPSPL6), which is connected to both central nodes (HPBAT1 and HHKO36P02G29PD). Interestingly, physical interaction between the two proteins ASK2 and CYC3B has been reported in *A. thaliana* (http://bar.utoronto.ca/interactions).

An enrichment analysis of GO terms associated with the nodes included in the network did not reveal over- or under-represented ontological terms (*p* < 0.05, Benjamini & Hochberg's FDR correction with significance level: 0.05).

**Figure 4:**
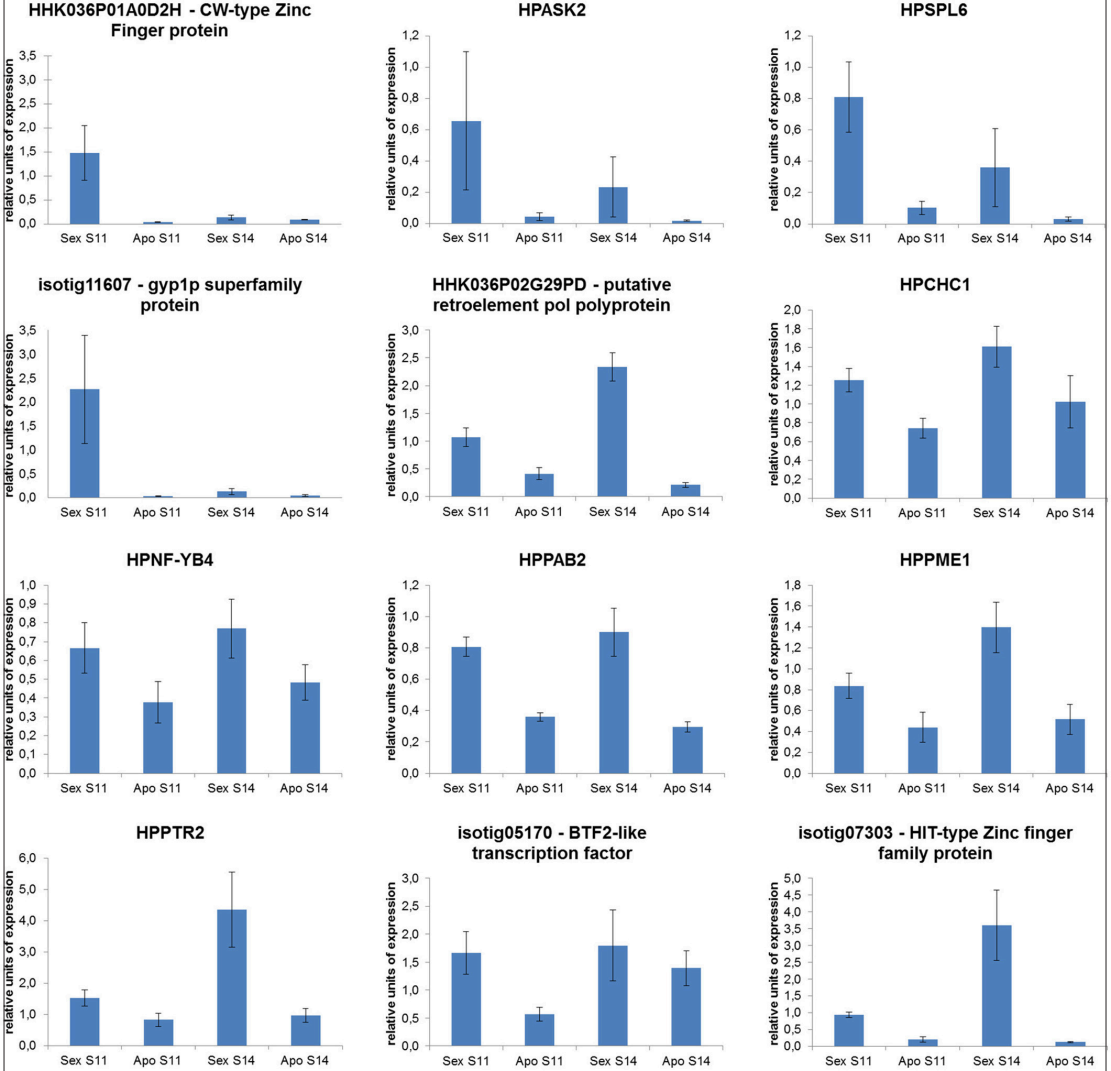
**Quantitative Real-Time PCR results for a number of ***H. perforatum*** genes included in the expression correlation sub-network potentially associated with the induction of the aposporous developmental programme**.

Sixteen genes were selected among those included in the expression correlation network (displayed in Figure 3 and Table 6) and their expression tested by Real-Time RT-qPCR assays. From this group of DEGs, 12 unigenes displayed a similar expression pattern as they were significantly down-regulated in aposporous pistils in correspondence of both developmental stages (Figure 4). In particular, this expression pattern was observed for the unigene HHKO36P01A0D2H, which shares some similarity with a MORC family CW-type zinc finger protein 4, the unigene isotig25064, which is predicted to encode a protein that belongs to the chromodomain remodeling complex (HPCHC1), and three unigenes encoding for transcription factors belonging to the families NF-YB4, BTF2-like, and HIT-type Zinc finger family protein (Table 6, Figure 4). The same expression pattern was observed for the two unigenes: HPPTR2 and HHK036P02G29PD (Figure 4), the latter of which encodes for putative retroelement pol protein (Table 6).

In contrast, the unigene HPBRI1 was down-regulated in aposporous pistils only in the terminal developmental stage (Figure S5). Finally, Real-Time RT-qPCR assays could not confirm the differential expression detected with the microarray analysis for the unigenes: HPALADIN (isotig13015), HPBAT1 (isotig18376), and HPCYC3B (HHKO36P02GL54X) (Figure S5 and Table S7).

## Discussion

### Expression analysis of sexual and apomictic *H. perforatum* genotypes reveals highly differentiated transcriptome signatures at early stages of pistil development

From the reproductive point of view, *H. perforatum* is an aposporous apomictic species whereby the alternative differentiation of a somatic cell gives rise to a functional embryo sac containing an unreduced egg cell. In principle, aposporous initial cells in apomictic plants are somatic cells belonging to the nucellus, which change their fate through the ability to mitotically divide and develop functional embryo sacs by mimicking sexual gametogenesis (Barcaccia et al., 2007; Galla et al., 2011). While the unreduced egg cell has the ability to undergo parthenogenesis, fertilization of the binucleated central cell of an aposporous embryo sac by a pollen nucleus is normally required for endosperm formation (Matzk et al., 2001). Expression analyses were performed on *Hypericum* pistils collected from genotypes exhibiting opposing reproductive forms at flower developmental stages spanning megasporogenesis and megagametogenesis. These developmental stages were selected to investigate expression changes associated with the transition from sporogenesis to gametogenesis in sexual pistils, concomitant to apomeiosis and development of aposporous initials in aposporous pistils.

Overall, considering the expression of all DEGs (Figure S1), apomictic samples were characterized by high variability in terms of the expression levels of multiple genes, leading to high differentiation of samples at both stages. Conversely, a principal component analysis revealed higher uniformity in sexual samples, whereby biological replicates clustered more closely together. Noticeably, restricting the analysis to unigenes which, based on their annotations, are potentially related to reproduction (Figure 1) allowed for a closer clustering of apomictic samples in correspondence of both developmental stages. High variability in overall gene expression observed for the aposporous pistils collected at stages corresponding to apomeiosis and development of the aposporous initials (Figure S1) might be reflective of the asynchronous development of these processes. The comparison between apomictic and sexual pistils at stage 14 underlined the relatively low number of genes that were significantly differentially expressed. Conversely, highly differentiated transcriptome signatures characterized sexual and apomictic pistils collected at flower stage 11. This flower developmental stage in apomictic genotypes encompasses the failure of the meiotic program and changes in cell fate required for the induction of aposporous embryo sac development. We are aware that the complexity of the analyzed tissue is such that multiple genes could be modulated in response to processes not directly connected to ovule and gamete development. Nevertheless, the switch from sexuality to aposporous apomixes, that is phenotypically characterized by dramatic changes in ovule and gamete development, was reflected in the modulation of the expression of 760 genes between sexual and aposporous samples. Remarkably, approximately 95% of genes modulated in this developmental stage were down-regulated in aposporous samples. This pervasive decrease in the expression of many genes could be reflective of a change in the rate or timing of development of some cell lines in the ovule relative to others (Galla et al., 2011). Temporal shifts in the expression of genes involved in the sexual developmental program have been already proposed in both diplosporous (Grimanelli et al., 2003) and aposporous (Galla et al., 2015) plant species. Interestingly, a global decrease in gene expression within the apomict compared to the sexual ovules during the early stages of development was also reported for the diplosporious *Boechera divaricarpa* (Sharbel et al., 2010). The extent to which de-regulation of genes required for sexual gametes formation is shared among aposporous and diplosporous species is currently not known. Nevertheless, this finding pinpoints an intriguing similarity in overall transcriptome changes occurring in concomitance with embryo sac development from somatic cells and unreduced functional megaspores, as observed in both aposporous and diplosporous species.

Ontological annotation of DEGs identified in each pairwise comparison led to the identification of several classes of annotation associated with flower development, the regulation of cell cycle progress and DNA metabolic processes. Along with these annotations, multiple terms were related to responses to biotic and abiotic stimuli and carbohydrate metabolic processes. Similarly, Schmidt et al. (2014) reported that microdissected aposporous initial cells of *Boechera gunnisoniana* were enriched for terms associated with carbohydrate transport, membrane lipid biosynthetic process and terms associated with the biosynthesis and transport of amino acids. Modulation of the genes involved in responses to biotic and abiotic stimuli as well as carbohydrate and lipid metabolisms in sexual vs. apomictic accessions was previously documented by Galla et al. (2015). Interestingly, although phenotypic changes associated with apomeiosis of the apospory-type are apparently restricted to a limited number of cells within the ovule, these highly localized developmental alterations are accompanied by the modulation of large transcriptome portions (Table 2), involving the sensing (i.e., response) of external (exogenous biotic or abiotic) and internal (i.e., endogenous) stimulus (Table 3) and the generation or catabolism of precursor metabolites and energy (Table 3). More recently, Klatt et al. (2016) observed that photoperiod extension, which could be regarded as a moderate abiotic stress, has a significant effect on the rate of megasporogenesis in the facultative aposporous apomictic *Ranunculus*. Future researches will be aimed at understanding if the failure of the meiotic program, the lack of a functional megaspore and/or the induction of apospory are sensed by the sporophytic cells embracing the gametophyte as internal stimuli, which in turn could directly or indirectly elicits wide transcriptional changes in the ovule.

### Induction of apospory is concomitant with the differential expression of key genes involved in the sexual reproductive pathway and RNA-directed DNA methylation

Multiple genes involved in female sporogenesis were significantly modulated throughout pistil development. Strikingly, the vast majority of sporogenesis-related DEGs were modulated in aposporous pistils during their development (cluster C4) and compared to their sexual counterparts (cluster C6) while only HPANT, the putative bipolar kinesin KRP-130 and HPRAD54 were down-regulated in sexual pistils (cluster C2).

To explain this finding, several scenarios can be taken into account. While it is possible that some genes not included in the array are involved in sporogenesis and gametogenesis, the present design of the array includes over 2500 unigenes that, based on their annotation, were predicted to be involved in ovule development and gamete formation (Galla et al., 2015). Otherwise, we could hypothesize a relatively low level of synchronism in sporogenesis progression alongside the proximal-distal axis of the pistil (Galla et al., 2011), leading to high variability within and among biological replicates and a concomitant decrease in our statistical power to detect significantly modulated genes. Interestingly, this hypothesis would imply that once established, transcriptional changes linked to the early events of aposporous apomixis (i.e., apomeiosis and differentiation of the aposporous initial) might be within view for a relatively long time.

**Figure 5:**
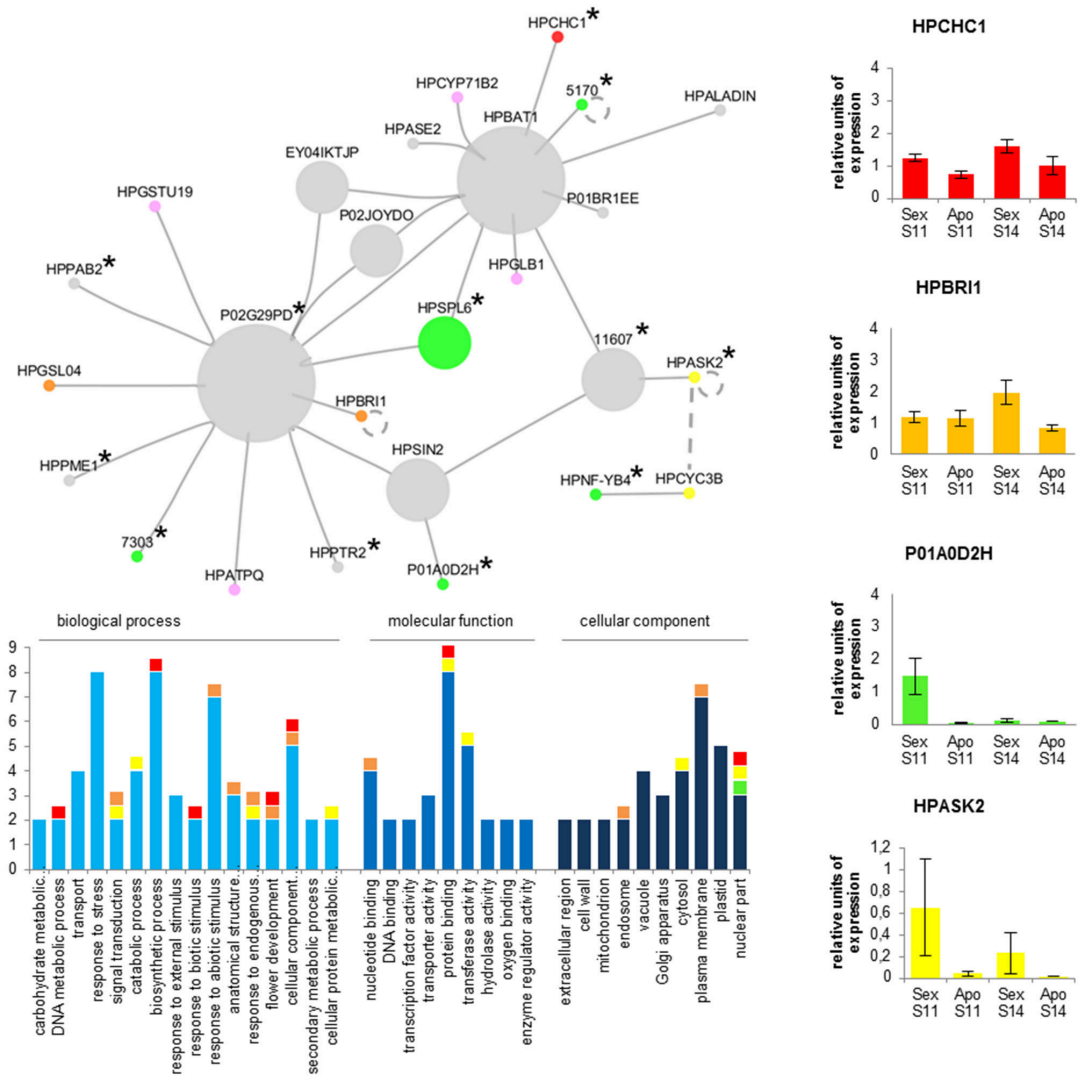
**(A)** Cytoscape visualization of the expression correlation sub-network generated by filtering all DEGs as: [(C1 OR C2) AND (C5 OR C6)]. Nodes size is proportional to number of connections. Green nodes, transcription factors; yellow nodes, cell cycle regulators; red node, chromatin remodeling factor; orange nodes, reproductive-related unigenes; pink nodes, stress related proteins; gray nodes, other annotations. Continuous lines indicate edges identified in the expression correlation network while dotted lines indicate known physical interactions between the *A. thaliana* orthologues. Unigenes whose expression has been validated by Real-Time qPCR are indicated with the asterisk (^*^). **(B)** Quantitative Real-Time PCR results for the unigenes included in the expression correlation network: HPCHC1, HPBRI1, P01A0D2H, and HPASK2. **(C)** Ontological annotation of unigenes included in the expression correlation network. Annotations of the unigenes whose expression is reported on **(B)** are indicated with colored squares. Red, HPCHC1; orange, HPBRI1; green, P01A0D2H; yellow, HPASK2.

Interestingly, the analysis of ontological terms significantly overrepresented in the cluster of genes down regulated in aposporous pistils at stage 14 (clusters C3 and C4) underlined a significant enrichment of terms associated with DNA binding and DNA-methyltransferase activity, which was not observed in sexual pistils collected at comparable developmental stages. Recently, Garcia-Aguilar et al. (2010) reported that the DNA methylation pathway active during reproduction is essential for gametophyte development in maize and likely plays a critical role in the differentiation between apomictic and sexual reproduction. In the same study, the authors observed that loss-of-function mutants for two genes involved in the maintenance of DNA-methylation status result in phenotypes that are strikingly reminiscent of apomictic development, suggesting that, in addition to a crucial role in gametophyte development, DNA methylation in the maize ovule might regulate the transcriptional expression of genes involved in the differentiation between apomixis and sexual reproduction (Garcia-Aguilar et al., 2010). Similarly, a study examining the distribution of cytosine methylation at the apomixis locus in two aposporous *Paspalum* species revealed that artificial demethylation induced a significant depression of parthenogenesis, suggesting that factors controlling the repression of parthenogenesis might be inactivated in apomictic *Paspalum* by DNA methylation (Podio et al., 2014). In this frame, it is worth noting that the two genes involved in the maintenance of DNA methylation at CpG and non-CpG sites: MEE57 (Borges and Martienssen, 2013) and CMT3 (Henikoff and Comai, 1998), decreased their expression throughout pistil development in aposporous but not in sexual samples. It is worth noting that CMT3 is a key determinant for CpNpG methylation in *A. thaliana* (Lindroth et al., 2001) and its expression is critical for the maintenance of methylation at transposon-related sequences (Kato et al., 2003). Furthermore, its expression is also required for H3K9 dimethylation in the egg cell and for normal embryogenesis during the first few divisions of the zygote (Pillot et al., 2010). Intriguingly, CMT3 is also involved in the RNA directed DNA methylation pathway in Arabidopsis (Kawashima and Berger, 2014), suggesting that differentiation of the aposporous initial might be somehow related to miss regulation of the RNA-directed DNA methylation (RdDM) pathway or improper spatial/temporal expression of its components. Further experimental evidence that RNA directed DNA-methylation changes might be critical for the switch from sexual to aposporous developmental pathways derives from the finding that an apospory-like phenotype is associated with *argonaute 9* (*ago9*) mutants in *Arabidopsis thaliana* (Olmedo-Monfil et al., 2010). AGO9 preferentially interacts with 24-nucleotide small interfering RNAs (siRNA) derived from transposable elements to direct homolog-based RNA-directed DNA methylation (RdDM). Moreover, mutations in SUPPRESSOR OF GENE SILENCING3 (SGS3) and RNA-DEPENDENT RNA POLYMERASE6 (RDR6), two genes required for siRNA biogenesis, also lead to a defect identical to that in ago9 mutants (Olmedo-Monfil et al., 2010). In a previous study, we demonstrated that the expression of HPAGO9 is significantly reduced in aposporous pistils at floral stage 11, while the expression of HPSGS3 and HPRDR6 has no or little variation between sexual and aposporous pistils (Galla et al., 2015). These findings were confirmed by the present expression analysis. Additionally, our microarray data and Real-Time qPCR assays indicated that the unigene isotig06679, which encodes for an orthologue of *Arabidopsis* INVOLVED IN *DE NOVO* 2 (IDN2), a double-stranded RNA-binding protein involved in *de novo* methylation and small interfering RNA (siRNA)-mediated maintenance of methylation (Finke et al., 2012), was significantly down-regulated in aposporous pistils at stage 11 compared to sexual pistils (FC: −10.42). Previously, Zhu et al. (2013) demonstrated that IDN2 binds to Pol V-produced noncoding RNAs and physically interacts with MORC family proteins and SWI3B, a subunit of the SWI/SNF chromatin-remodeling complex, thus contributing to noncoding RNA-mediated transcriptional silencing. Noteworthy, our microarray and Real-Time qPCR assays showed that the unigene HHKO36P01A0D2H, sharing high similarity with a MORC-like CW-type Zinc finger protein, is significantly down regulated in aposporous pistils at both floral developmental stages. Additionally, the expression of the unigene isotig25064, which is predicted to encode a SWI/SNF ASSOCIATED PROTEINS 73 (HPCHC1), was also found to be reduced in aposporous pistils (Table 6, Figure 4). The two unigenes: isotig25064 and HHKO36P01A0D2H were both included in the expression correlation sub-network (Table 6, Figures 3, 5) whose modulation in aposporous pistils is concomitant with apomeiosis and aposporous initial induction and development. Interestingly, Jégu et al. (2015) demonstrated that CHC1 controls cytokinin biosynthesis and cell cycle progression in the Arabidopsis root, by directly modulating the deposition of H3K27 and H3K4me3 histone marks as well as RNA Pol II occupancy at the IPT3 and IPT7 loci. Whether the same actors regulate the expression of HPIDN2 (unigene: isotig06679), the MORC-like CW-type Zinc finger protein (unigene: HHKO36P01A0D2H) and HPCHC1 (isotig25064) and what is nature of these regulators is currently elusive. Nevertheless, the finding that expression of HPCHC1 and the MORC-like CW-type Zinc finger protein is highly correlated with that of several transcription and cell cycle regulators (Table 6, Figure 4) might lead us to speculate that possible regulators or targets of the RdDM machinery in aposporous pistils could be included among these co-expressed genes.

### Unigenes involved in homeostasis, signalling and response to hormones are differentially expressed in aposporous pistils

Cytokinins and auxins are classes of plant hormones with a broad range of functions in regulating plant growth and development as well as physiological processes (Werner and Schmülling, 2009; Kieber and Schaller, 2014). It is known that activity of the two hormones in promoting stem cell maintenance or cell differentiation is defined based on the specific environmental context (Bencivenga et al., 2012). Key components of cytokinin biosynthesis and response modulators are expressed during ovule development (Bencivenga et al., 2012). Overall, the ontological term “cytokinin catabolic process” was found to be significantly enriched in *B. gunnisoniana* aposporous initial cells compared to the cell types comprising the mature gametophyte (Schmidt et al., 2014). The expression data collected in sexual and aposporous pistils indicates that several genes involved in the biosynthesis and perception of cytokinin stimuli are down-regulated in aposporous pistils. Among these genes, IPT2, a tRNA isopentenyl transferase whose mis-expression has been associated with meiotic defects in *Arabidopsis*, was down regulated in aposporous pistils. This finding is coherent with the observed reduction in the expression of HPCHC1 (FC: −2.27), which is known to control cytokinin biosynthesis by regulating the expression of several IPT genes.

Interestingly, among genes responsive to cytokine stimuli, the receptors and/or signal transducer ACR4 (Hsieh and Goodman, 2002) was down regulated (FC: −2.27) in aposporous pistils at stage 11. It has long been known that an abundance of the ACR4 transcript is increased in response to cytokinin (Hsieh and Goodman, 2002). It is worth noting that the expression of the homeodomain transcription factor BEL1, one of the major players in chalaza development, which is modulated in response to cytokinin levels in *Arabidopsis* (Bencivenga et al., 2012), was increased in *Hypericum* aposporous pistils at developmental stages spanning late gametogenesis by a fold change of 1.88.

As cytokinin was proposed to be involved in ovule development by modulating auxin fluxes through the control of PIN1 expression, we were also interested in the expression patterns of genes involved in auxin biosynthesis, homeostasis and responses. Among the auxin efflux carriers involved in ovule intercellular auxin gradients and maxima, such as PIN1, no significant changes in expression were observed in sexual and/or aposporous pistils. The same was true for the unigenes encoding HPPIN3 and HPPIN5. Instead, Real-Time qPCR assays revealed that the endoplasmic reticulum-localized HPPIN8 is up regulated in aposporous pistils at flower stage 11 and 14. Following the publications of Ding et al. (2012) and Dal Bosco et al. (2012), we have indications that PIN8 is involved in the regulation of intracellular auxin homeostasis and affects auxin-regulated gene transcription by either preventing access of auxin to the nucleus or altering the biologically active auxin pool within the nucleus.

Taken together, the present data indicate that modulation in the expression of genes involved in cellular auxin homeostasis, either by intracellular transport or hydrolysis of IAA-conjugates, is concomitant with aposporous gametophyte development. The finding that sexual gametophyte development is not associated with modulation of the same genes might lead to speculate a possible role for these proteins in modulating IAA cellular levels in ovules undergoing the aposporous developmental program. Accordingly, several genes regulated by auxin, some of which are known to be involved in meiosis and/or gametogenesis, were differentially expressed in our samples. Among these genes, the expression of ARF2, ARF7, a SAUR-like auxin-responsive family protein (homologous to AT5G20820), TRN2, the RNA polymerase II C-terminal domain phosphatase-like 2 (CPL2) and the SKP1-like protein 1B (ASK2) was significantly lower in the aposporous than the sexual pistils. Conversely, the auxin-responsive protein IAA4 and the SAUR-like auxin-responsive protein SAUR20 were up regulated in aposporous pistils (stage 14 vs. stage 11).

## Concluding remarks

Expression analyses were aimed at investigating expression changes associated with the transition from sporogenesis to gametogenesis in sexual pistils, concomitant with apomeiosis and development of aposporous initials in aposporous pistils. Data collected are coherent with the asynchronous or heterochronic expression of multiple genes in aposporous pistils, a phenomenon which, for the most part, could be restricted to the stage corresponding to apomeiosis and development of the aposporous initials. Ontological annotation of DEGs led to the identification of several classes of annotation associated with the regulation of cell cycle and DNA metabolic processes along with multiple terms related to responses to biotic and abiotic stimuli (Table 3, Figure 5). The three genes involved in the RdDM pathway: HPCHC1, IND2, and CMT3 along with a MORC-like protein likely involved in the same biological process, were significantly down regulated in aposporous pistils. This finding provides further evidences supporting the already proposed idea that phenotypic expression of apomixis is dependent upon the chromatin state in sporophytic cells embracing the gametophyte (Garcia-Aguilar et al., 2010). Furthermore, the annotation of unigenes included in the expression correlation network reported in Figure 5 provides evidences for a possible link between genes involved in the RdDM pathway (i.e., HPCHC1), stress responses and flower development. Remarkably, a number of genes involved in hormones perception and homeostasis, including cytokinin, auxin, and brassinosterois, were differentially expressed in aposporous pistils. The connection between hormones and the switch from the sexual to the aposporous developmental programs is further strengthened by the finding that cytokinin biosynthesis in Arabidopsis is regulated by the activity of CHC1 (Jégu et al., 2015). These findings provide new evidences in support of the idea that chromatin state in sporophytic cells embracing the gametophyte might influence the cellular levels of plant hormones and ultimately, based on the specific environmental context, modulate the expression of a wide range of genes, including reproductive and cell fate related genes, in response to internal or external stimuli.

## Author contributions

GB and GG conceived the study. SZ and LAV performed the Array design, cDNA synthesis and hybridization. GG carried out the computational analysis, validated the expression data and drafted the manuscript. LAL helped to annotate the *Hypericum* unigenes. All authors helped to draft the manuscript. All authors read and approved the final manuscript.

### Conflict of interest statement

The authors declare that the research was conducted in the absence of any commercial or financial relationships that could be construed as a potential conflict of interest.
