# Supplementary figures and images for: Pistil Transcriptome Analysis to Disclose Genes and Gene Products Related to Aposporous Apomixis in Hypericum perforatum L
Source: Front Plant Sci. 2017 Feb 1;8:79. doi: 10.3389/fpls.2017.00079 (PMC5285387; doi:10.3389/fpls.2017.00079)

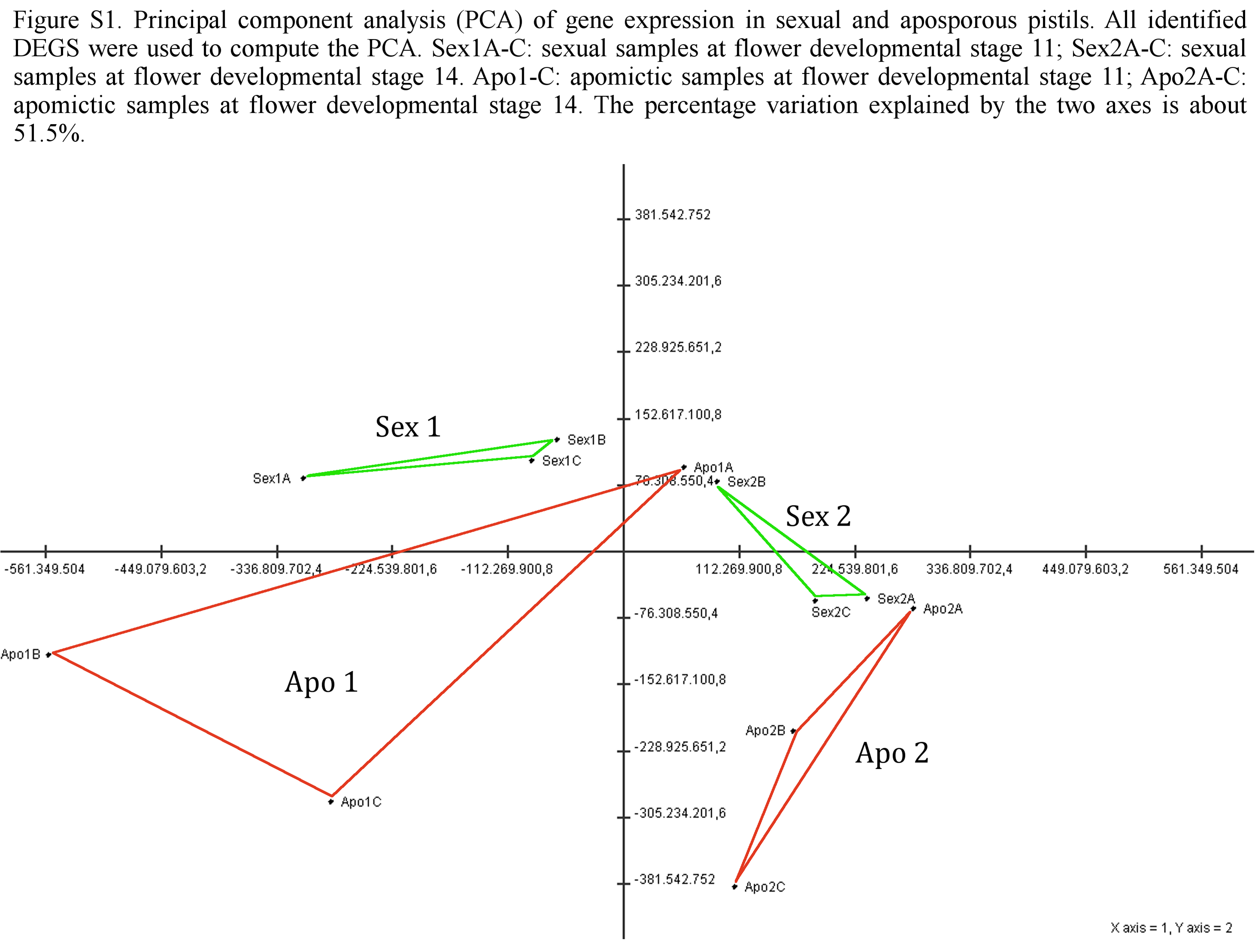

Supplement: Supplementary file 8 [file Image1.TIFF]

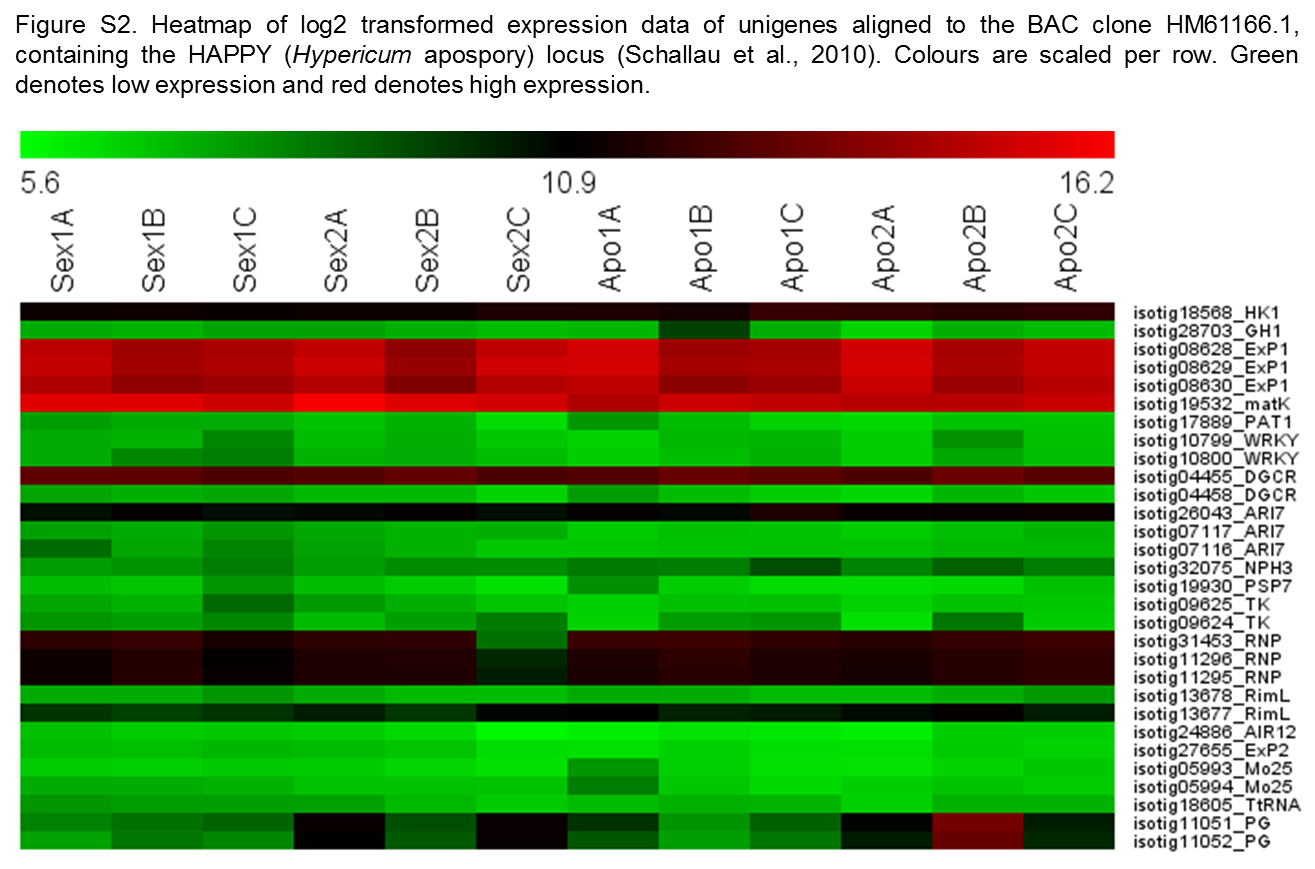

Supplement: Supplementary file 9 [file Image2.TIFF]

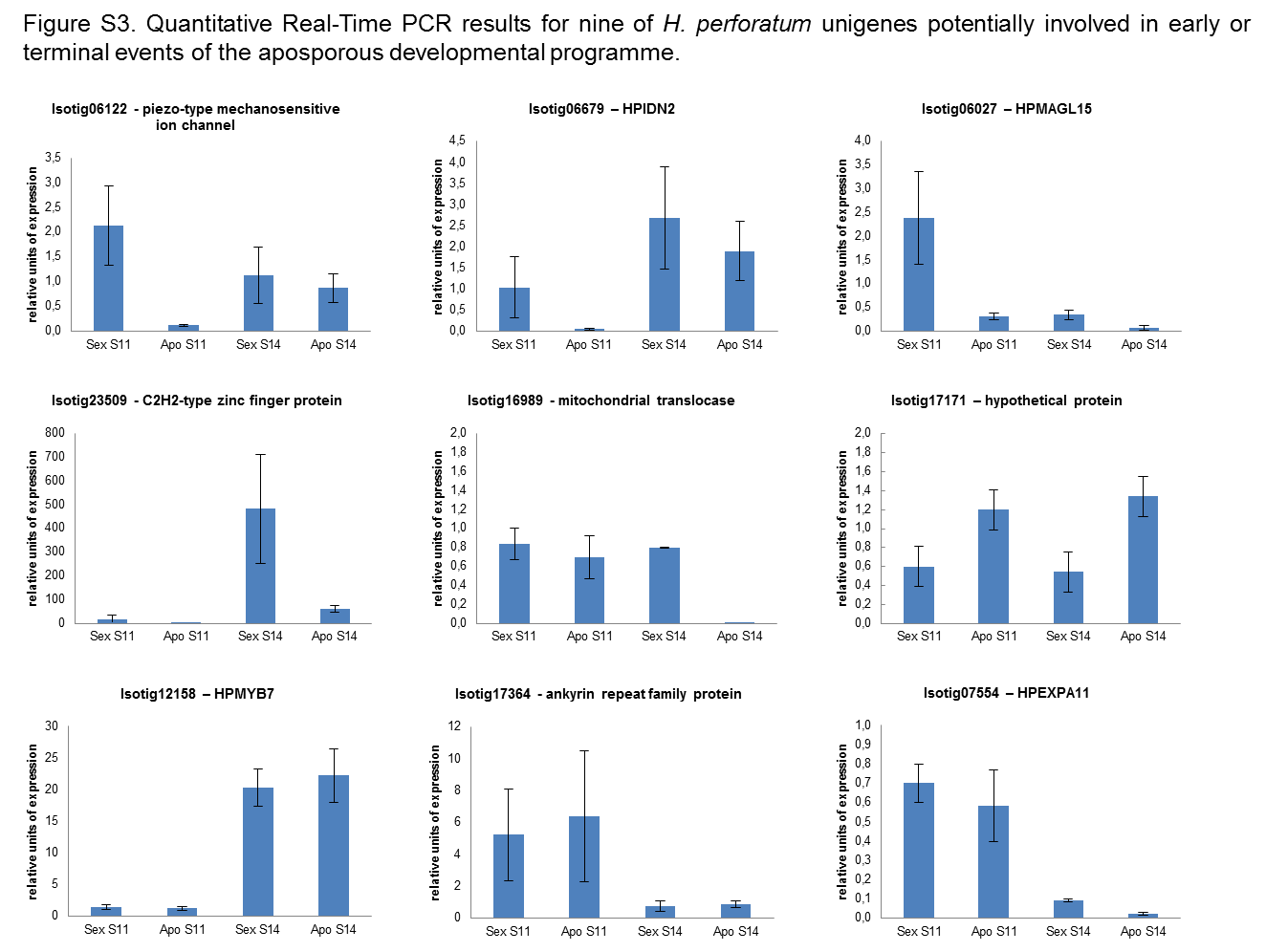

Supplement: Supplementary file 10 [file Image3.TIFF]

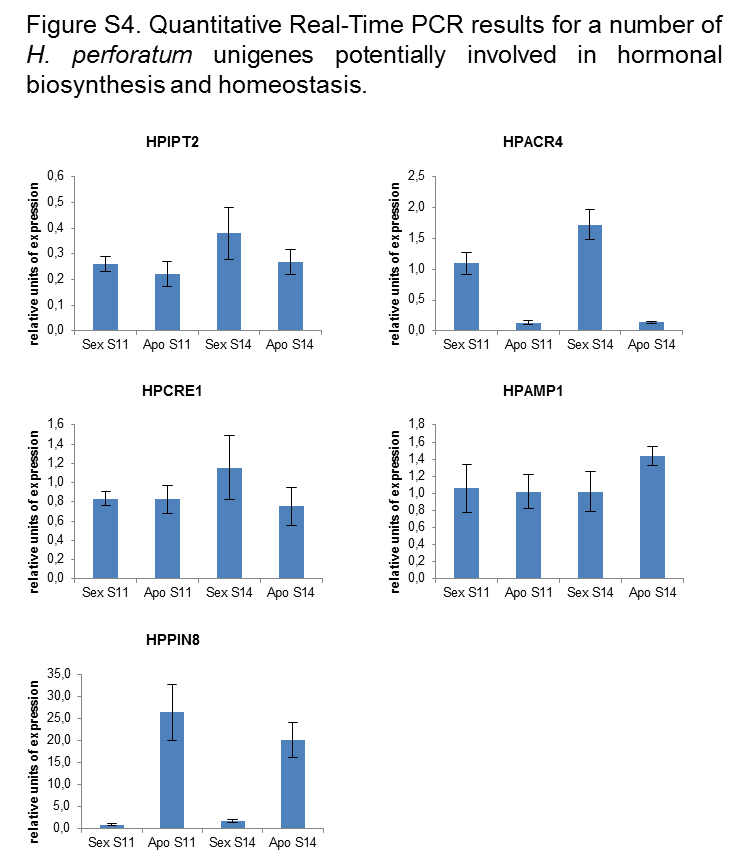

Supplement: Supplementary file 11 [file Image4.TIFF]

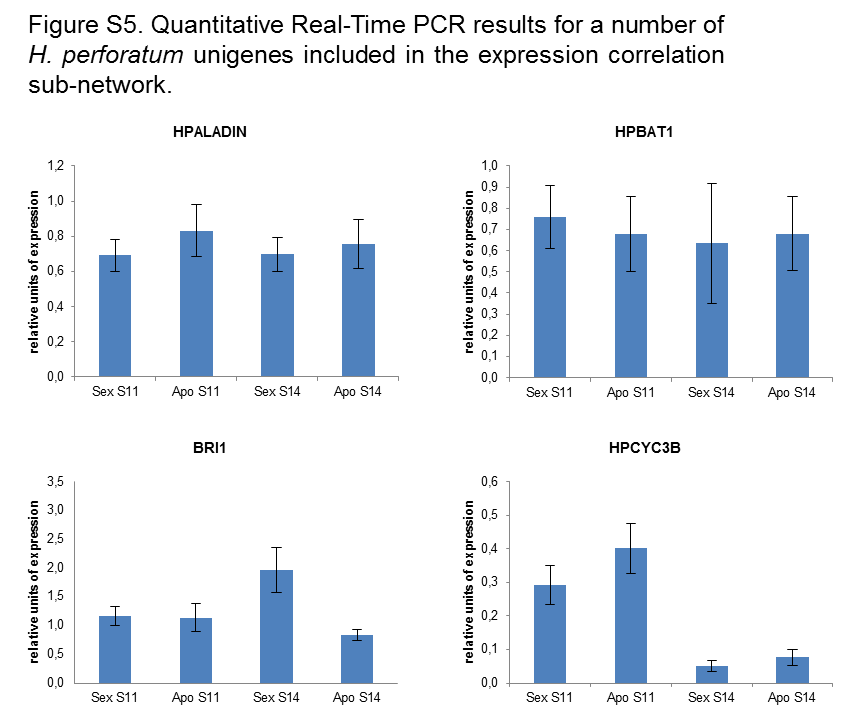

Supplement: Supplementary file 12 [file Image5.TIFF]
